# *Dlec1* is required for spermatogenesis and male fertility in mice

**DOI:** 10.1038/s41598-020-75957-y

**Published:** 2020-11-03

**Authors:** Yu Okitsu, Mamoru Nagano, Takahiro Yamagata, Chizuru Ito, Kiyotaka Toshimori, Hideo Dohra, Wataru Fujii, Keiichiro Yogo

**Affiliations:** 1 Department of Applied Life Sciences, Faculty of Agriculture, Shizuoka University, Shizuoka, Japan; 2 Department of Agriculture, Graduate School of Integrated Science and Technology, Shizuoka University, Shizuoka, Japan; 3 Department of Reproductive Biology and Medicine, Graduate School of Medicine, Chiba University, Chiba, Japan; 4 Research Institute of Green Science and Technology, Shizuoka University, Shizuoka, Japan; 5 Department of Animal Resource Sciences, Graduate School of Agricultural and Life Sciences, The University of Tokyo, Tokyo, Japan; 6 College of Agriculture, Academic Institute, Shizuoka University, Shizuoka, Japan

**Keywords:** Reproductive biology, Differentiation

## Abstract

Deleted in lung and esophageal cancer 1 (*DLEC1*) is a tumour suppressor gene that is downregulated in various cancers in humans; however, the physiological and molecular functions of *DLEC1* are still unclear. This study investigated the critical role of *Dlec1* in spermatogenesis and male fertility in mice. *Dlec1* was significantly expressed in testes, with dominant expression in germ cells. We disrupted *Dlec1* in mice and analysed its function in spermatogenesis and male fertility. *Dlec1* deletion caused male infertility due to impaired spermatogenesis. Spermatogenesis progressed normally to step 8 spermatids in *Dlec1*^*−/−*^ mice, but in elongating spermatids, we observed head deformation, a shortened tail, and abnormal manchette organization. These phenotypes were similar to those of various intraflagellar transport (IFT)-associated gene-deficient sperm. In addition, DLEC1 interacted with tailless complex polypeptide 1 ring complex (TRiC) and Bardet–Biedl Syndrome (BBS) protein complex subunits, as well as α- and β-tubulin. DLEC1 expression also enhanced primary cilia formation and cilia length in A549 lung adenocarcinoma cells. These findings suggest that DLEC1 is a possible regulator of IFT and plays an essential role in sperm head and tail formation in mice.

## Introduction

Spermatogenesis is a complex process in which spermatogonial stem cells differentiate into haploid sperm^1^. Spermatogonia proliferate by mitosis and differentiate into spermatocytes, which undergo two consecutive meiotic divisions to produce round spermatids. These round spermatids further undergo dynamic morphological and structural changes, such as elongation of the nucleus, condensation of chromatin, and formation of a flagellum, to form mature sperm. Many genes expressed in a spatially and temporally specific manner regulate these changes^2,3^. However, the molecular function of each gene is still unclear.

In the later phase of spermatogenesis, two microtubule-based structures, axoneme and manchette, are involved in sperm flagella formation and head shaping^4^. The axoneme is a core structure in the flagellum composed of nine outer doublet microtubules and two central microtubules (9 + 2 structure) attached with many structural components, such as an inner dynein arm (IDA), an outer dynein arm (ODA), a radial spoke, and the nexin–dynein regulatory complex (N-DRC)^5^. This structure is highly conserved in the motile cilia of somatic cells, except in nodal cilia, which have a 9 + 0 structure similar to non-motile primary cilia. During spermatogenesis, the axonemal microtubules act as a platform for intraflagellar transport (IFT)^4^. IFT is a bidirectional intracellular trafficking system necessary for cilia/flagella formation. Since cilia/flagella do not contain ribosomes, the proteins and membrane necessary to build a flagellum are transported from the base of the flagellum to the tip by the motor protein kinesin (anterograde transport), while unnecessary proteins are returned to the base by dynein (retrograde transport)^6,7^. IFT-A, IFT-B, and Bardet–Biedl syndrome proteins (BBSome) play an important role in IFT. These are large protein complexes comprising many subunits and are involved in the recognition of cargo and loading on the motor protein^8^. IFT-A and IFT-B bind to dynein and kinesin, respectively, and are involved in retrograde and anterograde transport, respectively. BBSome binds to cargo proteins and act as mediators of cargo loading onto the IFT complex. However, the regulation of the formation of these complexes is still unclear.

The manchette is a microtubule-/F-actin-containing skirt-like structure that transiently appears around the caudal region of nuclei and flagella in elongating spermatids. Although findings about the nucleation site and the direction of microtubules are inconsistent^9,10^, microtubules extend from the perinuclear ring (a portion that corresponds to the skirt waist and exists just below the acroplaxome marginal ring) into the cytoplasm and/or basal body of the spermatid. During spermatid differentiation, the manchette moves toward the tail neck region, with constriction of the perinuclear ring, which contributes to sculpting of the sperm head in mice. Similar to the axoneme, proteins are actively transported on manchette microtubules via intramanchette transport (IMT)^4^. IMT shares similar molecular components as IFT, including motor proteins and IFT protein complex.

Deletion of IFT-/IMT-associated genes significantly affects sperm morphogenesis and male fertility. Lehti et al.^11^ reported that *Kif3a* deletion in the germ cell lineage severely impairs flagella formation, nuclear remodelling, and manchette organization in mice. Similarly, several IFT complex subunit-deficient male mice (IFT20^12^, IFT25^13^ IFT27^14^ and IFT140^15^) also shows infertility due to impaired spermatogenesis with sperm head and flagella malformation.

Recessive mutation in the oligotriche locus (*olt*) causes male fertility in the mouse^16^. Homozygous mutant (*olt/olt*) mice show alopecia in the inguinal region and male infertility due to impaired spermatogenesis. In *olt/olt* mice, spermatogenesis is impaired after meiosis, and the number of sperm at step 13 or later is significantly lower compared to wild type (WT) mice. Additionally, *olt/olt* mice do not have flagella in the seminiferous lumen, indicating impaired flagellum development. Runkel et al.^17^ reported that *olt/olt* mice have a 234 kbp deletion in distal chromosome 9. This region contains six genes: *carboxy-terminal domain small phosphatase-like protein* (*Ctdspl*), *villin-like* (*Vill*), *1-phosphatidylinositol-4,5-bisphosphate phosphodiesterase delta-1* (*Plcd1*), *Dlec1*, *acetyl-coenzyme A acyltransferase 1B* (*Acaa1b*), and *solute carrier 22a member 14* (*Slc22a14*). Of these, *Plcd1* and *Acaa1b* are not required for male fertility in mice, indicating that *Ctdspl*, *Vill*, *Dlec1*, and *Slc22a14* are candidate genes responsible for male infertility. Slc22a14, a member of the organic anion/cation transporter family, plays a critical role in male infertility^18^. However, spermatogenesis in *Slc22a14*-knockout (KO) mice is essentially normal. Therefore, a gene essential for male fertility other than *Slc22a14* should be present in the *olt* locus; however, no reports showing that *Ctdspl* or *Vill* is involved in spermatogenesis or flagellar formation are available.

*DLEC1* (also called *DLC1*) was originally identified as a gene that is downregulated in human lung and esophageal cancers^19^. Decreased *DLEC1* expression is found in various cancers; hypermethylation of the *DLEC1* promotor region causes this decreased expression^20–24^. Introduction of the DLEC1 expression vector into cancer cells or treatment with 5-aza-2′-deoxycytidine, which induces DNA demethylation, inhibits cell proliferation and/or malignancy^23,25,26^, so DLEC1 is believed to act as a tumour suppressor. *FAP81*, an ortholog gene of *Dlec1* in *Chlamydomonas reinhardtii*, is found as a flagellar protein using proteomic analysis of isolated flagella^27^. Zhao et al.^28^ found that FAP81 is a novel central apparatus protein in *Chlamydomonas*, and Fu et al.^29^ showed that FAP81 is required for flagellar motility and assembly of the C1e-c complex that attaches to the central microtubule pair. In addition, DLEC1 has plural ASPM-SPD-2-Hydin (ASH) domains that are involved in protein–protein interactions and are often found in cilia/flagella or centrosome-associated proteins in mammalian cells^30,31^. These findings suggested that DLEC1 is involved in cilia/flagella formation or function, in addition to inhibition of tumorigenesis.

This study investigated the expression profiles of *Dlec1*, *Vill*, and *Ctdspl* for infertility in *olt/olt* male mice, with an emphasis on *Dlec1*. We disrupted *Dlec1* in mice and analysed its function in spermatogenesis and male fertility.

## Results

### *DLEC1* is expressed in spermatids and spermatozoa

**Figure 1:**
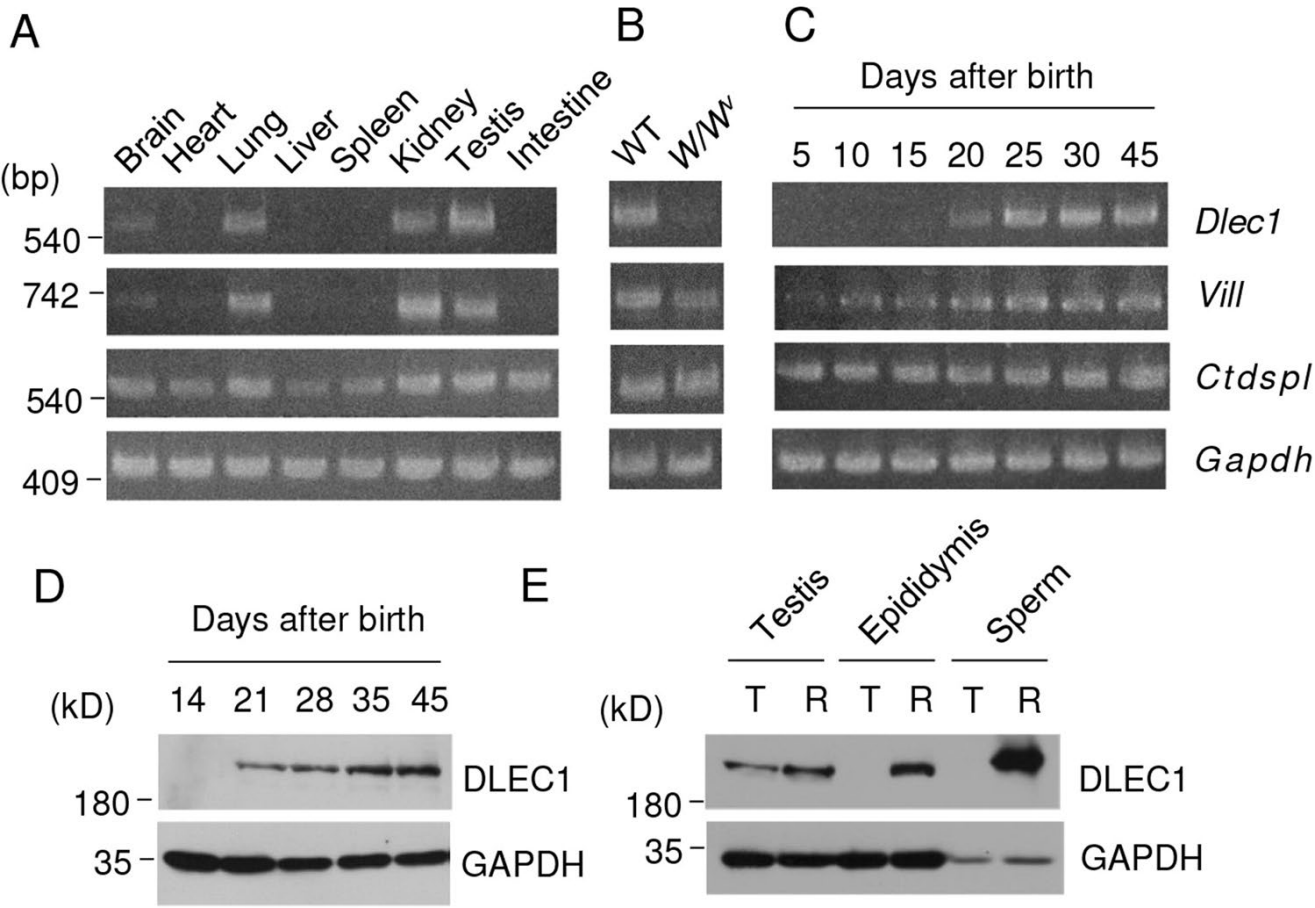
Expression analysis of mouse DLEC1. (**A**–**C**) Analysis of *Dlec1*, *Vill*, and *Ctdspl* expression using RT-PCR. Expression in (**A**) various mouse tissues, (**B**) WT and *W/W*^V^ mutant mouse testes, and (**C**) testes during first-wave spermatogenesis. The numbers indicate days after birth. (**D**) DLEC1 expression during postnatal testicular development was examined by western blotting. The numbers indicate days after birth. (**E**) DLEC1 expression in testes, cauda epididymis, and cauda epididymal sperm. 1% Triton X-100-containing lysis buffer (T) or 0.1% SDS-containing RIPA buffer (R) was used to solubilize the tissue and use for western blotting. Images of full-length gels and immunoblots are presented in the supplementary Fig. S6.

Toward the identification of the gene responsible for male infertility in *olt/olt* mice, we investigated the expression profiles of candidate genes (*Dlec1*, *Vill*, and *Ctdspl*) in various mouse tissues using reverse transcription-polymerase chain reaction (RT-PCR). As shown in Fig. 1A, *Dlec1* and *Vill* were expressed in the brain, lungs, kidneys, and testes, while *Ctdspl* showed a broad expression pattern. We investigated the expressions in *W/W*^v^ mouse testes which lack germ cells because of the mutation on c-kit^32^ to elucidate whether these genes are specifically expressed in germ cells. Expression of *Ctdspl* and *Vill* were observed in *W/W*^v^ mouse testes, and expression of *Dlec1* was not detected, indicating that only *Dlec1* among these genes is specifically expressed in germ cells in the testes (Fig. 1B).

Next, we investigated *Dlec1*, *Vill*, and *Ctdspl* expression during first-wave spermatogenesis. *Vill* was expressed at PND (postnatal day) 5 and reached a maximum at PND 20–25. *Ctdspl* was expressed constantly during testicular development, while *Dlec1* expression began at PND 20 and plateaued at PND 25 (Fig. 1C). DLEC1 protein expression also showed a similar pattern (Fig. 1D). DLEC1 expression was also detected in the cauda epididymis and cauda epididymal sperm (Fig. 1E). Since haploid spermatids first appear at 18 days after birth^33^, these results indicated that DLEC1 expression starts in round or early elongating spermatids. Interestingly, although DLEC1 could be solubilized in the testes with either 1% Triton X-100-containing lysis buffer or radioimmunoprecipitation assay (RIPA) buffer, it could be solubilized in the epididymis and sperm only with RIPA buffer, which has stronger protein solubilization ability (Fig. 1E).

### *Dlec1* is indispensable for spermatogenesis and male fertility

To investigate the physiological role of *Dlec1*, we developed *Dlec1*^*−/−*^ mice using the Clustered Regularly Interspaced Short Palindromic Repeats and CRISPR-associated (CRISPR-Cas9) system (Supplementary Fig. S1A). *Dlec1* deletion was confirmed by genome PCR (Supplementary Fig. S1B). Although we detected expression of *Dlec1* messenger RNA (mRNA) that lacks exons 28–33 (~ 830 bp) in homozygous mutant mice (Supplementary Fig. S1C), this mRNA does not seem to be translated efficiently. The predicted short form of the protein (~ 157.1 kDa, DLEC1ΔC) in *Dlec1*^*−/−*^ testes could be detected, but the expression level was approximately one-tenth expression of wild-type DLEC1 (Supplementary Fig. S1D). Although DLECΔC potentially acts as a dominant negative mutant because it lacks the third ASH domain (Supplementary Fig. S1E), the fertility and spermatogenesis of heterozygous mutant male mice was normal. Thus, we considered DLEC1ΔC does not play a substantial role in vivo. Validation of the anti-mouse DLEC1 antibody we developed is shown in Supplementary Fig. S2. In addition, we did not detect off-target effects for at least two potential off-target sites for each guide RNA (Supplementary Fig. S3).

**Figure 2:**
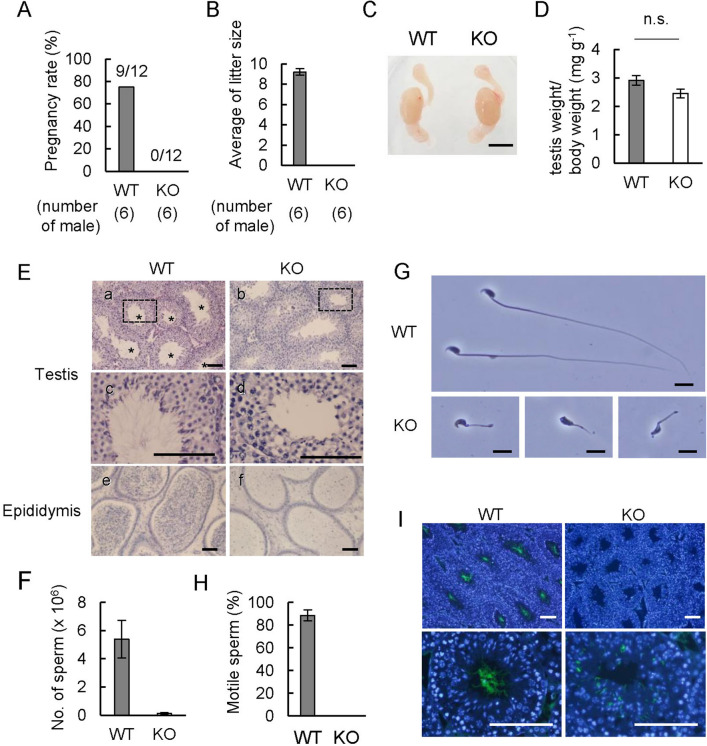
*Dlec1* is required for spermatogenesis and male fertility. Each male mouse was caged with two WT BDF1 female mice for 2 weeks, and the (**A**) pregnancy rate (number of pregnant females/number of females mated) and (**B**) average of litter size were measured (*n* = 6). (**C**) Appearance of testes and epididymides of WT and *Dlec1*^*−/−*^ mice. (**D**) Testis weight per body weight of WT and *Dlec1*^*−/−*^ mice (*n* = 6). No significant statistical difference between WT and *Dlec1*^*−/−*^ mice was detected (*p* = 0.066). (**E**) Sections of testis (a–d) and cauda epididymis (e and f) were stained using H&E. *Seminiferous tubule where sperm flagella were observed. (c and d) Magnified areas of the boxes in (a) and (b), respectively. Scale bar = 100 µm. (**F**) Number of cauda epididymal sperm in WT and *Dlec1*^*−/−*^ mice (*n* = 5). (**G**) Morphology of WT and *Dlec1*^*−/−*^ sperm. Scale bar = 10 µm. (**H**) Percentage of motile sperm of WT and *Dlec1*^*−/−*^ sperm (*n* = 5). Error bars indicate standard error. (**I**) The testis section was stained using anti-acetylated tubulin antibody (green) and DAPI (blue). No acetylated tubulin signal was observed in the seminiferous tubule lumen in *Dlec1*^*−/−*^ testes. Scale bar = 100 µm.

*Dlec1*^*−/−*^ mice were born at the expected Mendelian ratio, and visual examination showed their growth and behaviour to be normal. In contrast, the mating experiment showed that *Dlec1*^*−/−*^ male mice are infertile. In WT pair mating, 75% of female mice delivered, with an average litter size of 9.2 ± 0.32 (Fig. 2A,B), while no pups were born when *Dlec1*^*−/−*^ male mice were mated with WT female mice (Fig. 2A,B). The frequency of plug formation was the same for WT and KO mice. Although the gross appearance (Fig. 2C) and weight of testes (Fig. 2D) were comparable between WT and homozygous mutant mice, histomorphological analysis revealed that spermatogenesis is impaired in *Dlec1*^*−/−*^ mice. Although we found spermatogonia, spermatocytes, and round spermatids in *Dlec1*^*−/−*^ seminiferous tubules, mature sperm were hardly observed (Fig. 2E). The number of cauda epididymal sperm was extremely low in *Dlec1*^*−/−*^ mice (Fig. 2E,F). Sperm collected from the cauda epididymides of *Dlec1*^*−/−*^ mice showed an abnormal head shape and short flagellum (Fig. 2G) and were immotile (Fig. 2H). Impaired flagella formation in *Dlec1*^*−/−*^ mice was also confirmed by immunofluorescence analysis using anti-acetylated tubulin antibody, which is known as a flagella marker (Fig. 2I).

**Figure 3:**
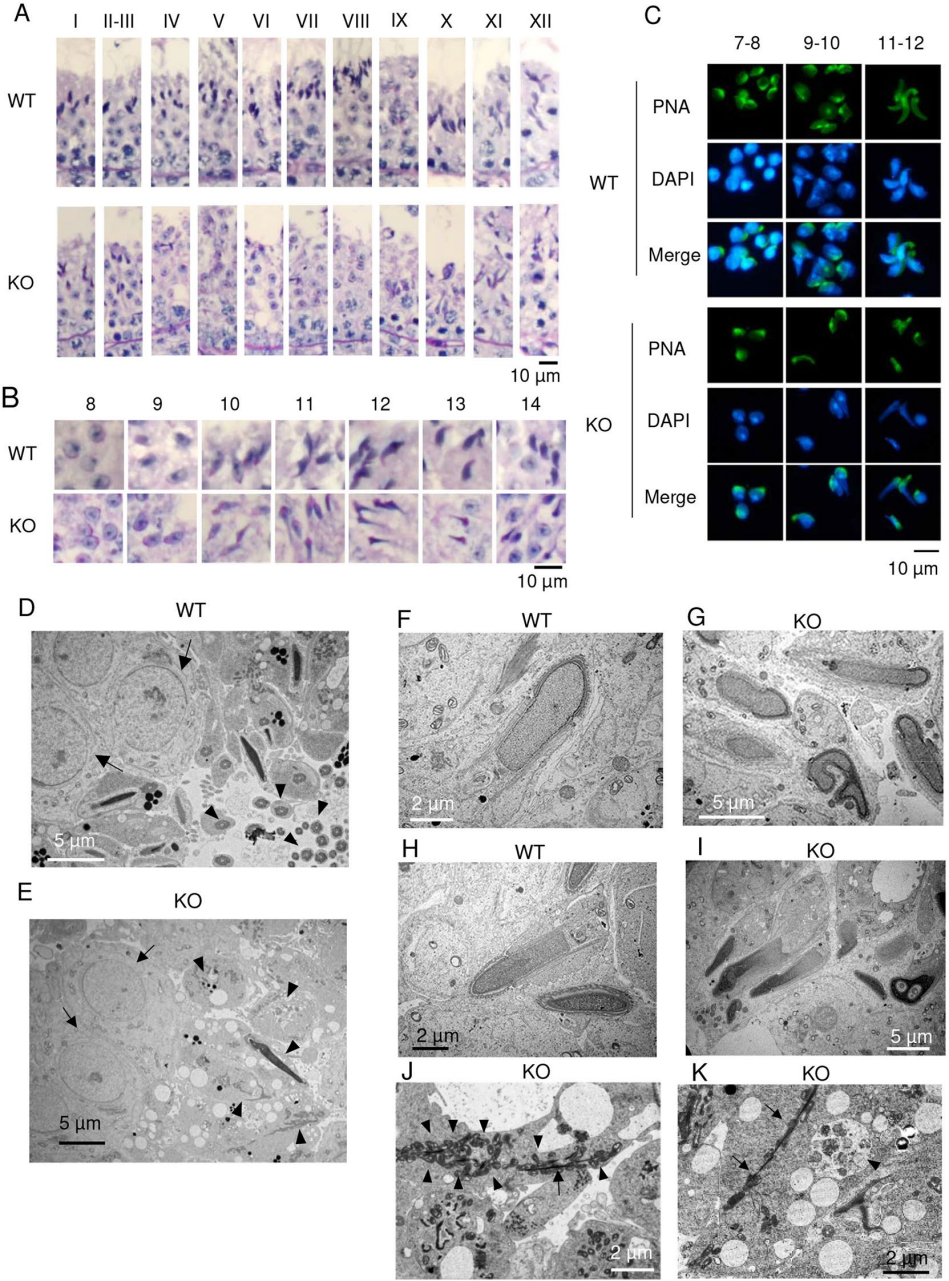
Spermatogenesis is impaired at the elongating spermatid stage in *Dlec1*^*−/−*^ mice. (**A**) Seminiferous epithelium in WT and *Dlec1*^*−/−*^ mouse testis. Testis sections were stained using periodic acid solution and haematoxylin. Roman numerals indicate stages of the cycle. (**B**) Spermatids at each stage of spermatogenesis in WT and *Dlec1*^*−/−*^ mice. Testis sections were stained as in (**A**). Numerals indicate the stages of spermatids. (**C**) Acrosome formation in WT and *Dlec1*^*−/−*^ spermatids. Isolated spermatids (step 7–12) were stained with Alexa488-conjugated peanut agglutinin (PNA, green). (**D**–**K**) Electron microscopy of WT and *Dlec1*^*−/−*^ testis cross sections. (**D**) Seminiferous tubule at stage VI in WT testis. Arrows and arrowheads indicate round spermatids and flagella cross sections, respectively. (**E**) Seminiferous tubule at stage VII in *Dlec1*^*−/−*^ testis. Although round spermatids are normal (arrow), many degenerated cells and irregular cellular components were observed (arrowhead). (**F**–**I**) Elongating spermatids (step 11 and 12) in (**F**,**H**) WT and (**G**,**I**) *Dlec1*^*−/−*^ testes at (**F**,**G**) stage XI and (**H**,**I**) stage XII. The head shape was deformed in *Dlec1*^*−/−*^ spermatids. (**J**) Abnormal tail-like structure in *Dlec1*^*−/−*^ spermatids. Misaligned mitochondria (arrowhead) and fibre-like structures (arrow) were observed. (**K**) Phagocytosis of degenerated spermatids by Sertoli cells. Digested cellular components in the phagosome (arrowhead) and incorporation of the fibre-like structure (arrow) were observed.

We investigated spermatogenesis in *Dlec1*^*−/−*^ mice more precisely. Although spermatogenesis was normal until steps 8 and 9 of spermatids, sperm head deformation became apparent after step 10 (Fig. 3A,B). The number of spermatids decreased as spermatogenesis proceeded in the cycle of the seminiferous epithelium, and at step 14 or later, we hardly observed any spermatids. This phenotype was very close to that of *olt/olt* mice^17^. We further investigated acrosome morphology of *Dlec1*^*−/−*^ spermatids by staining with Alexa488-conjugated peanut agglutinin (Fig. 3C). The acrosome appeared to be normally formed until round spermatid in *Dlec1*^*−/−*^ mice; however, it's shape gradually deformed as nuclei deformed during differentiation. Acrosomes were observed in the dorsal region of the sperm head in wild-type spermatids at step 11–12, but such polar localisation was not clearly observed in *Dlec1*^*−/−*^ spermatids. The sperm head and flagella abnormality was confirmed by electron microscopy (Fig. 3D–K). Consistent with the results of light microscopy analysis, round spermatids of *Dlec1*^*−/−*^ at stage VII look healthy (Fig. 3D,E, arrow). In WT testes, we observed many flagella cross sections in the same seminiferous tubule (Fig. 3D, arrowhead). In contrast, in *Dlec1*^*−/−*^ testes, we observed many degenerated cells and irregular cellular components, such as nuclei, acrosomes, and flagella (Fig. 3E, arrowhead). In elongating spermatids, most of the nuclei were distorted or irregular in *Dlec1*^*−/−*^ testes (Fig. 3F–I). We could not find an axonemal structure despite an extensive search. Instead, misaligned mitochondria were frequently observed around a fibre-like structure in *Dlec1*^*−/−*^ elongating spermatids (Fig. 3J). Since these degenerated cellular components appeared to be incorporated into Sertoli cells (Fig. 3K), the decreased number of sperm in *Dlec1*^*−/−*^ testes was probably because of phagocytosis by Sertoli cells.

### *Dlec1*^*−/−*^ sperm has an abnormal manchette structure

**Figure 4:**
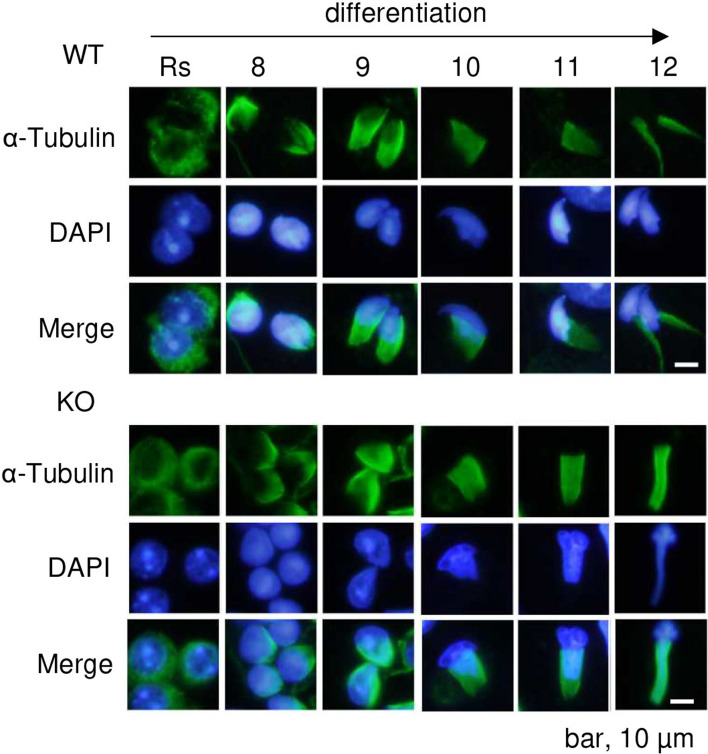
The manchette is abnormally elongated in *Dlec1*^*−/−*^ spermatids. Testicular cells were isolated and stained using anti-α-tubulin antibody (mouse monoclonal, green). Photographs of each step of spermatids of WT and *Dlec1*^*−/−*^ mice were taken. Rs, round spermatids. Nuclei were stained using DAPI (blue).

Head deformation and impaired flagella formation in sperm are often accompanied by an abnormal manchette structure. We investigated the manchette structure by immunofluorescence analysis using anti-α-tubulin antibody. Although the shape of the *Dlec1*^*−/−*^ sperm manchette was normal in round and early elongating spermatids (steps 8–9), it abnormally elongated as spermatogenesis proceeded (Step 10–12) (Fig. 4); 4′,6-diamidino-2-phenylindole (DAPI) staining confirmed the abnormal head shape of *Dlec1*^*−/−*^ spermatids. Since these phenotypes of *Dlec1*^*−/−*^ sperm were similar to those of various IFT-associated gene-KO sperm, such as *Kif3a*^11^, we believed *Dlec1* deletion to affect those expressions. However, KIF3A, IFT25 (a component of the IFT-B complex), and IFT140 (a component of the IFT-A complex) expression in testes was not different between WT and *Dlec1*^*−/−*^ mice (Supplementary Fig. S4).

### Identification of DLEC1-interacting proteins

To determine the molecular function of DLEC1, we identified DLEC1-interacting proteins. Since commercially available or custom-made anti-DLEC1 antibodies are not suitable for immunoprecipitation of endogenous DLEC1, we introduced 3 × FLAG-tagged human DLEC1 (hDLEC1) into human embryonic kidney 293F (HEK293F) cells, and proteins that co-immunoprecipitated with DLEC1 were identified by liquid chromatography–tandem mass spectrometry (LC–MS/MS) (Supplementary Fig. S5 and Supplementary Table S1). In all, 67 proteins were identified, including many cilia-/flagella-associated proteins, such as α-tubulin, β-tubulin, and T-complex protein 1 (TCP-1) family proteins (Table 1). α- and β-Tubulin are the main structural components of axonemes in cilia and flagella. TCP-1 forms a complex called tailless complex polypeptide 1 ring complex (TRiC) or chaperonin-containing TCP-1 (CCT), which acts as a chaperon for tubulin and actin^34^. In addition to being a chaperon, TRiC enhances retrograde axonal transport in neurons^35^, forms a complex with BBS6, BBS10, and BBS12, and mediates BBSome assembly^36^. BBSome is a complex of seven BBS proteins (BBS1, BBS2, BBS4, BBS5, BBS7, BBS8, and BBS9) and is involved in the recognition and trafficking of cargo in or to cilia and flagella.

**Table 1:** DLEC1-interacting proteins predicted to be associated with flagella formation.

Accession no	Protein name	− 10lg*P*	Coverage (%)	Peptides	Function (reference)
NP_001257329.1	Tubulin alpha-1A chain isoform 2	200.5	44	18	Component protein of the axoneme
NP_006073.2	Tubulin alpha-1B chain	199.4	40	18	
NP_001290045.1	Tubulin alpha-1C chain isoform b	205.0	49	19	
NP_821133.1	Tubulin beta chain isoform b	219.8	43	23	
NP_821080.1	Tubulin beta-2B chain	216.1	43	23	
NP_006077.2	Tubulin beta-3 chain isoform 1	204.0	34	18	
NP_006079.1	Tubulin beta-4B chain	220.0	43	23	
NP_115914.1	Tubulin beta-6 chain isoform 1	181.2	33	16	
NP_110379.2	T-complex protein 1 subunit alpha isoform a	60.8	8	4	Cytoplasmic chaperon for microtubule^34^, enhancement of retrograde axonal transport^35^, mediation of assembly of BBSome^36^
NP_001185771.1	T-complex protein 1 subunit beta isoform 2	114.0	16	7	
NP_006421.2	T-complex protein 1 subunit delta isoform a	109.1	16	7	
NP_036205.1	T-complex protein 1 subunit epsilon isoform a	83.9	13	7	
XP_011530780.1	T-complex protein 1 subunit eta isoform X1	63.1	4	2	
NP_001008800.1	T-complex protein 1 subunit gamma isoform c	90.7	11	5	
NP_006576.2	T-complex protein 1 subunit theta isoform 1	148.8	24	12	
NP_001753.1	T-complex protein 1 subunit zeta isoform a	86.6	11	6	
NP_005336.3	Heat shock 70 kDa protein 1A	254.6	37	25	Molecular chaperon localised in the flagella^27,46^
XP_011541100.1	Heat shock cognate 71 kDa protein isoform X1	224.9	34	21	Molecular chaperone that interacts with IFT88 and is involved in a formation or stabilization of IFT-cargo complexes^54^
NP_001308119.1	ruvB-like 2 isoform 2	132.9	21	8	An AAA ATPases compose R2TP complex which requires for axonemal dynein assembly^55^
NP_001306013.1	ruvB-like 1 isoform 2	65.5	12	4	
NP_002697.1	Protein phosphatase 1B isoform 1	150.5	32	9	A family of protein Ser/Thr phosphatases that localised in sperm flagella^47^
NP_001243728.1	Glyceraldehyde-3-phosphate dehydrogenase isoform 2	100.3	10	2	Glycolytic enzyme localised in the axoneme^27^

3 × FLAG-tagged hDLEC1 was expressed and immunoprecipitated with anti-FLAG antibody. Co-immunoprecipitated proteins were identified by LC–MS/MS analysis. Proteins known to be localised in the axoneme of cilia/flagella or predicted to be involved in cilia and/or flagella formation/function were selected. All proteins identified are shown in Supplementary Table S1. The accession number, protein name, − 10 × log10(*P*-value) (− 10lg*P*), percentage of protein sequence coverage, number of peptides identified, and function of the protein are shown.

**Figure 5:**
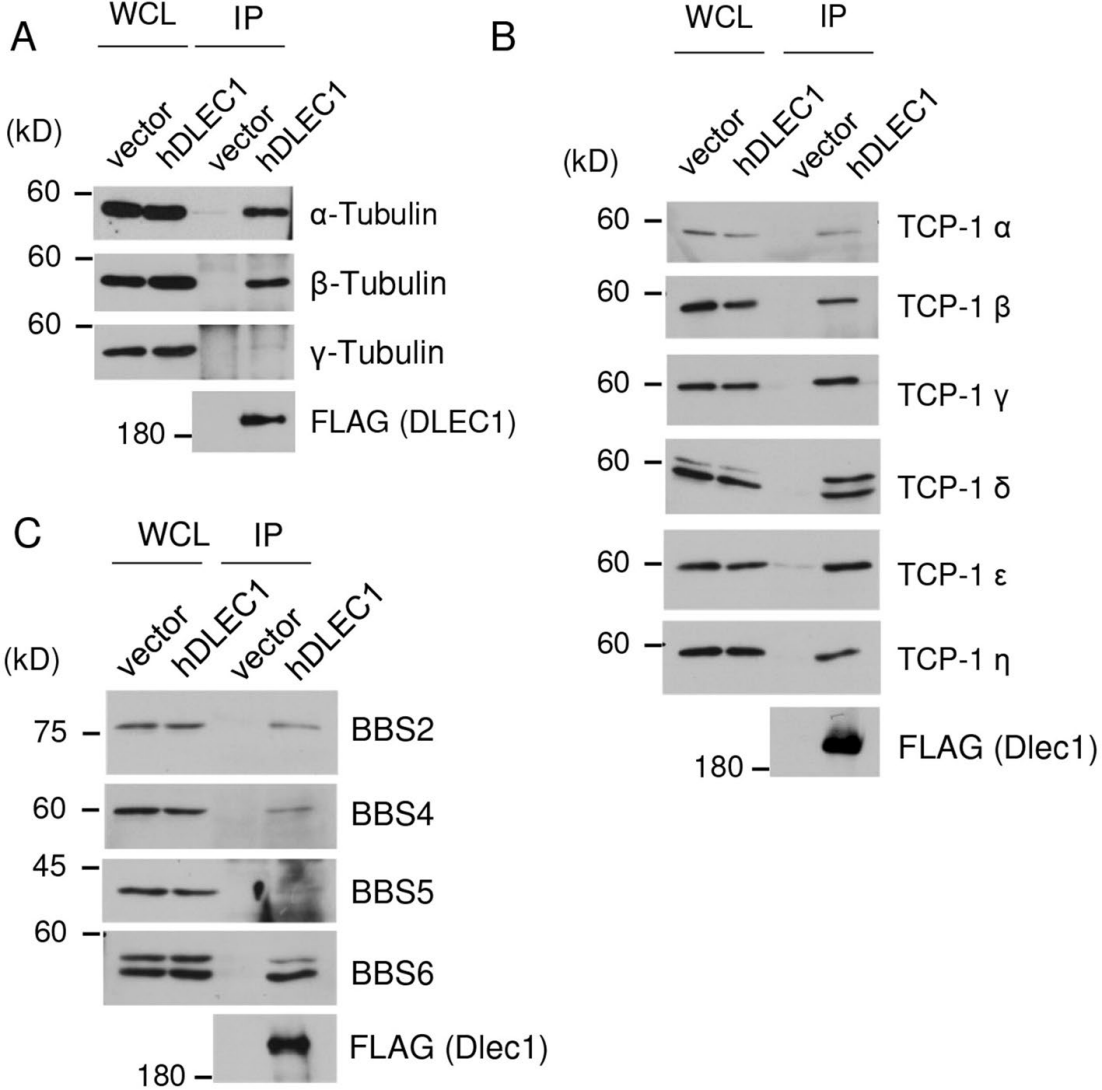
DLEC1 interacts with tubulin, TRiC subunits, and BBSome subunits. 3 × FLAG-tagged human DLEC1 (FLAG-hDLEC1) or empty vector was expressed in HEK293F cells and immunoprecipitated using anti-FLAG antibody. SDS-PAGE was applied to the immunoprecipitates, followed by western blotting using (**A**) tubulin antibodies, (**B**) TRiC subunit antibodies, and (**C**) BBSome subunit antibodies. WCL, whole cell lysate. Images of full-length immunoblots are presented in the supplementary Fig. S7.

We confirmed the interaction of hDLEC1 with α- and β-tubulin in HEK293F cells by immunoprecipitation–western blotting (Fig. 5A). However, hDLEC1 did not bind to γ-tubulin efficiently (Fig. 5A). We also detected the interaction of hDLEC1 with TRiC subunits (TCP-1 α, TCP-1 β, TCP-1 γ, TCP-1 δ, TCP-1 ε, and TCP-1 η) (Fig. 5B). In addition, although LC–MS/MS could not identify BBSome subunits, hDLEC1 interacted with BBS2, BBS4, BBS5, and BBS6 (Fig. 5C).

### DLEC1 expression enhances ciliogenesis in A549 cells

**Figure 6:**
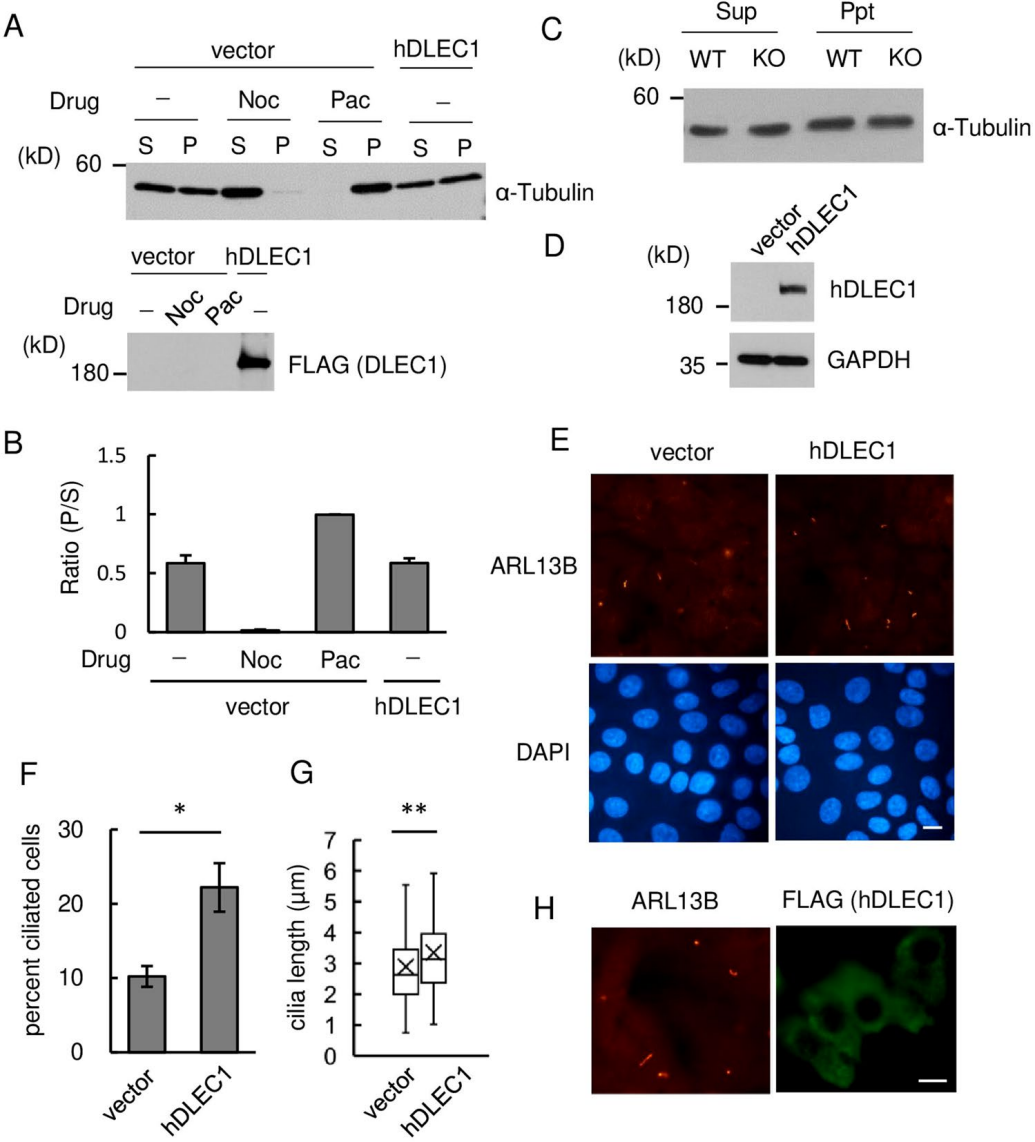
DLEC1 expression enhances primary cilia formation. (**A**) Upper panel: Tubulin polymerization assay. FLAG-hDLEC1 was introduced into HEK293F cells, and the cells were lysed with hypotonic buffer. After centrifugation, supernatants (S) and precipitates (P) were collected and used for western blotting. Nocodazole (Noc) and paclitaxel (Pac), which induce microtubule destabilization and stabilization, respectively, were used as a control experiment. Lower panel: DLEC1 expression was confirmed by western blotting. (**B**) Quantification results of tubulin polymerization assay shown in (**A**). P/S indicates ratio of signal intensity of precipitates (P) and supernatants (S). Data are presented as average ± standard error (*n* = 3). (**C**) Tubulin polymerization in WT and *Dlec1*^*−/−*^ testicular cells was monitored as in (**A**). (**D**) Establishment A549 cells stably express human DLEC1. Empty vector or 3 × FLAG-tagged human DLEC1 expression vector were introduced into A549 cells and selected by puromycin. Expression of DLEC1 was monitored by western blotting using anti-human DLEC1 antibody. (**E**) Empty vector or hDLEC1 expressing A549 cells grown to confluent were serum-starved for 24 h and stained with anti-ARL13B antibody, a primary cilia marker. Scale bar = 10 μm. (**F**) Percentage of cells with cilia were quantified. Over 500 cells in one experiment were counted, and data were shown as average ± standard error (*n* = 3). Asterisk indicates a significant difference (Student’s *t* test, *p* < 0.05). (**G**) Ciliogenesis was induced as described in (**E**) and cilia length were measured. Data were counted from approximately 300 cilia from 3 independent experiments and shown in boxplots. Double asterisk indicates significant differences (Student’s *t* test, *p* < 0.01). (**H**) Intracellular localisation of hDLEC1 in A549 cells. 3 × FLAG-tagged hDLEC1 expressing A549 cells were immunostained with anti-ARL13B and anti-FLAG antibodies. Images of full-length immunoblots are presented in the supplementary Fig. S8.

The above results suggest that DLEC1 is involved in flagella formation through regulation of microtubule polymerization and/or intracellular vesicle trafficking in or to flagella. Initially, we investigated the effects of hDLEC1 expression on tubulin polymerization in HEK 293F cells using tubulin polymerization assay. DLEC1 expression did not affect tubulin polymerization (Fig. 6A,B). We also investigated the amount of polymerized tubulin in mouse testes and found that it was not different between WT and *Dlec1*^*−/−*^ testes (Fig. 6C). This result is consistent with previous results that show that the manchette itself is formed in *Dlec1*^*−/−*^ spermatids (Fig. 4). Next, we investigated the effect of expression of DLEC1 on primary cilia formation in A549 lung adenocarcinoma cells. These cells show ciliogenesis induced by serum starvation. We established a cell line stably expressing hDLEC1 (Fig. 6D) and immunostained cells with anti-ARL13B antibody, a marker of primary cilia (Fig. 6E). Quantification of results revealed that DLEC1 expression increased the percentage of ciliated cells (Fig. 6F). In addition, DLEC1 expression slightly but significantly increased ciliary length, although it is unclear whether the difference has functional meaning in the cilia (Fig. 6G). We also investigated intracellular localisation of DLEC1. DLEC1 appeared to be localized relatively uniformly in cytoplasm or as indistinct dot-like structures, but not to primary cilia (Fig. 6H). These results suggest that DLEC1 may regulate protein transport from cytoplasm to primary cilia rather than within primary cilia.

### *Dlec1* deletion does not affect TRiC and BBSome expression, complex formation, and localisation

**Figure 7:**
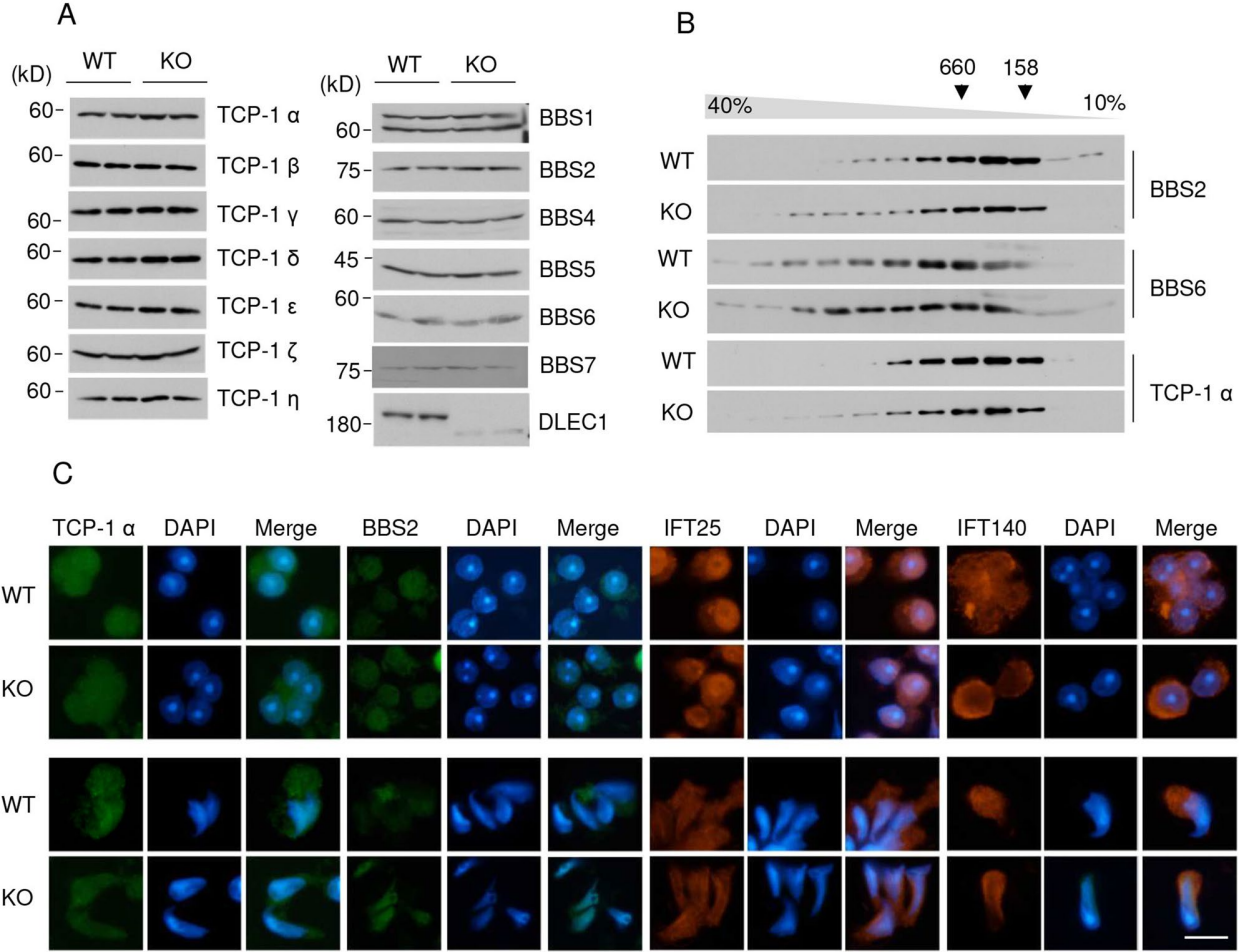
Expression, complex formation, and intracellular localisation of TRiC and BBSome is normal in *Dlec*^*−/−*^ testis. (**A**) TRiC and BBSome subunit expression in WT and *Dlec1*^*−/−*^ testes was monitored by western blotting. (**B**) Complex formation of TRiC and BBSome in WT and *Dlec1*^*−/−*^ testes. Testis lysates were layered onto a 10–40% sucrose gradient and centrifuged at 28,000 rpm for 16 h. The fractions were recovered and western blotting applied. Positions of molecular weight standards are indicated at the top. (**C**) Intracellular localisation of TCP-1 α, BBS2, IFT25, and IFT140 in WT and *Dlec1*^*−/−*^ round (upper) and elongating spermatids (lower). Scale bar = 10 μm. Images of full-length immunoblots are presented in the supplementary Fig. S9.

Next, we investigated the effect of *Dlec1* deletion on TRiC and BBSome subunit expression, complex formation, and intracellular localisation. The expression of each TCP-1 and BBsome subunit in *Dlec1*^*−/−*^ testis was comparable to that of WT testis (Fig. 7A). Complex formation of TRiC and BBSome in WT and *Dlec1*^*−/−*^ testes were investigated using sucrose density gradient centrifugation. BBS2, BBS6, and TCP-1-α form a complex of ~ 670–880 kDa, 770–1000 kDa, and 770–1000 kDa, respectively, in the testes^36^, which was roughly similar to our result; however, there was no apparent difference in complex formation between WT and *Dlec1*^*−/−*^ testes (Fig. 7B). Protein complex formation of TCP-1 alpha and BBS2 appears to be slightly different in KO mice, but results were not reproducible.

Intracellular localisation of TCP-1α, BBS2, IFT25, and IFT140 were investigated by immunofluorescence staining. Signals of these proteins in round spermatids were observed in the cytoplasm, and some were also seen in nuclei (Fig. 7C). These proteins in elongating spermatids were mainly localized posterior of nuclei (presumably manchette) in wild type. These staining patterns in *Dlec1*^*−/−*^ spermatids were essentially equivalent to the wild type; although the cell morphology and cell nuclei were abnormal, the proteins appeared to be mainly localized to manchette. These results suggested that DLEC1 regulates functions of IFT-related protein but not its expression, complex formation, or intracellular localisation.

## Discussion

This study revealed that *Dlec1* is essential for spermatogenesis and male fertility in mice. *Dlec1* is specifically expressed in germ cells in the testes, and *Dlec1* expression increases during spermatogenesis. Although spermatogenesis in *Dlec1*^*−/−*^ mice appears normal until step 8–9 spermatids, sperm head deformation is observed in subsequent stages of spermatogenesis. In addition, the tail of *Dlec1*^*−/−*^ sperm is short, and we could not find normal axonemal structures. The number of mature sperm is extremely low compared to WT mice, and *Dlec1*^*−/−*^ male mice are infertile. These abnormalities are closely similar to those of *olt/olt* mice. The oligotriche locus contains the *Slc22a14* gene that plays a critical role in male fertility. Since spermatogenesis in *Slc22a14*^*−/−*^ testis was normal, lack of the gene would not contribute to impaired spermatogenesis in *olt/olt* mice. In fact, spermatogenic phenotype of *Slc22a14/Dlec1* double KO mice was the same as *Dlec1* single KO mice (data not shown). Therefore, the lack of *Dlec1* is probably responsible for infertility in *olt/olt* male mice, though careful investigation for the involvement of *Ctdspl* and *Vill* is necessary*.* In addition to sperm head and flagella malformation, *Dlec1*^*−/−*^sperm have an abnormally elongated manchette, which is frequently observed in mice sperm that lack microtubule-regulating protein, such as KATNB1 (a subunit of the microtubule-severing complex)^37^, leucine-rich repeats and guanylate kinase domain-containing isoform 1 (LRGUK-1)^38^, HOOK1^39^, and cytoplasmic linker protein of 170 kDa (CLIP-170; the microtubule plus-end-tracking protein)^40^. Similarly, KO mice do not have IFT machinery proteins, such as KIF3A^11^, IFT20^12^, IFT25^13^, IFT27^14^, IFT140^15^, BBS2^41^, BBS4^42^, BBS6^43^, and BBS7^44^ show abnormal sperm head and flagella. These phenotype similarities suggest that DLEC1 is involved in the microtubule-associated function. In fact, we found that DLEC1 can interact with many microtubule-/IFT-associated proteins, such as α-tubulin, β-tubulin, TRiC subunits, and BBSome subunits in HEK 293F cells. Moreover, the expression of DLEC1 enhances primary cilia formation in A549 lung cancer cells. This result suggests that DLEC1 may regulate IFT through interacting proteins. However, since DLEC1 expression suppresses cancer cell proliferation and formation of cilia is induced by arrest of cell growth^45^, the possibility that DLEC1 indirectly increased cilia formation in A549 cells cannot be excluded. Moreover, DLEC1 is unlikely to be an essential role in the formation of primary cilia in vivo; *Dlec1*^*−/−*^ mice did not show any ciliopathic disorder. Further, the functional significance of the interaction of DLEC1 and IFT-associated proteins need to be defined.

In this study, we did not obtain any direct evidence that DLEC1 is involved in regulating microtubule polymerization or IFT. DLEC1 expression does not affect tubulin polymerization, and IFT25, IFT140, and KIF3A expression is the same in WT and *Dlec1*^*−/−*^ testes. Similarly, there is no difference in protein expression, complex formation, and intracellular localisation in TRiC and BBSome between WT and *Dlec1*^*−/−*^testes, indicating that DLEC1 is not involved in the regulation of microtubule polymerization and IFT machinery formation. One possible function of DLEC1 is the loading of cargo proteins on the IFT machinery. We identified some proteins other than IFT machinery as DLEC1-interacting proteins, such as heat shock proteins, glyceraldehyde-3-phosphate dehydrogenase (GADPH), and protein phosphatase 1B (formerly called PP2CB). These proteins are localised in the flagella of *Chlamydomonas*^27,46^ and mammalian sperm^47^. Therefore, DLEC1 might mediate the loading of these proteins on the IFT machinery, promoting flagella formation. Otherwise, DLEC1 itself could be a cargo protein transported by the IFT machinery into the flagella. Although intracellular DLEC1 localisation in sperm is not identified, FAP81 is localised to the central apparatus in *Chlamydomonas* flagella^29^. In addition, DLEC1 in sperm is more resistant to extraction by Triton X-100 compared to DLEC1 in testes that contain more immature germ cells. Since axonemal proteins, such as RSPH6A and acetylated tubulin, are fractionated to the Triton-insoluble fraction in mouse sperm^48^, our results might indicate that DLEC1 is transported from the cytosol to flagella as spermatogenesis proceeds. Regarding this, although we found exogenously expressed DLEC1 is not localized to primary cilia in A549 cells, it may not reflect the intracellular localisation in sperm because primary cilia do not have central pair of microtubules. Anyway, it is apparent that elucidation of the intracellular localisation of DLEC1 in sperm is critical for verifying our hypothesis. In this study, despite several attempts, we could not produce an antibody suitable for immunostaining. We also developed knock-in mice, which express FLAG-tagged DLEC1, and applied to immunohistochemical study, but specific signal was not detected in sperm. It may be due to low expression levels of endogenous DLEC1 is low. It is necessary to develop another tagged DLEC1 knock-in mice which enable higher sensitive detection.

Cryoelectron tomography shows that the *Chlamydomonas* FAP81 mutant lacks central apparatus projections C1e and C1c. Since the C1e-c subcomplex size is estimated to be 1.2 MDa^29^, the assembly of many proteins could be disturbed because of a lack of FAP81. In fact, the amount of FAP92 and FAP216 decreases to 37% and 0.07%, respectively, in the FAP81 mutant. Therefore, it is plausible that DLEC1 is involved in protein assembly in the central apparatus projection subcomplex of the mammalian sperm axoneme. However, one cannot simply apply the results in *Chlamydomonas* to mammalian sperm. The phenotypes of the FAP81 mutant and *Dlec1*^*−/−*^ sperm are slightly different. The FAP81 mutant can swim but slowly, while *Dlec1*^*−/−*^ sperm are immotile. In addition, FAP81 mutation does not affect the length of flagella in *Chlamydomonas*, and ortholog genes of FAP92 and FAP216 are not found in mammals^28^. Therefore, DLEC1 might play another role, in addition to the protein assembly of the central apparatus projection subcomplex.

In summary, we identified *Dlec1* as an essential gene for spermatogenesis and male fertility in mice. In *Dlec1*^*−/−*^ mice, the number of sperm is extremely low, accompanied by head and tail malformation. The human *DLEC1* is a possible candidate for causative genes for idiopathic azoospermia or teratozoospermia. *DLEC1* is located at 3p22-p21.3 in humans, which is frequently missing in various tumours, indicating that this chromosome region is more unstable compared to other regions. Azoospermic men have a 2.2-fold higher cancer risk compared to non-azoospermic men^49^. Since many reports suggest that DLEC1 acts as a tumour repressor, this study might provide a clue to elucidating the link between azoospermia and cancer.

## Methods

### cDNA synthesis, genomic DNA isolation, and PCR

Total RNA was isolated from mouse tissues using ISOGEN (Nippon Gene, Tokyo, Japan) and used as a template for reverse transcription. Complementary DNA (cDNA) synthesis was performed using ReverTra Ace (TOYOBO Co., Ltd., Osaka, Japan) according to the manufacturer’s instructions. Mouse genomic DNA was isolated from the tail tip using standard phenol/chloroform extraction. Polymerase chain reaction (PCR) was performed using KOD Fx Neo DNA polymerase (TOYOBO). Supplementary Table S2 lists the primers used for PCR.

### Antibodies

Anti-mouse DLEC1 antibody was developed by immunization of recombinant mouse DLEC1 protein (1–300 aa) to rabbit. After immunization six times, serum was collected, and the antibody was affinity-purified by a column coupled with mouse DLEC1 recombinant protein. Immunization and serum collection were performed by Medical and Biological Laboratories Co., Ltd. Validation results for this antibody are provided in Supplementary Fig. S2. Supplementary Table S3 lists the other primary antibodies used and the concentration of use.

### *Dlec1*^*−/−*^ mice generation

*Dlec1*^*−/−*^ mice were generated using the CRISPR-Cas9 system, as previously described^50^. Two target sequences for *Dlec1* (5′-GGGGTGACCTTGACCGTAGTGGG-3′ and 5′-CCTCTGCACACGCTCGACACAGCC-3′) were selected to eliminate exons 28–33, and these DNA fragments were inserted into the plasmid vector containing guide RNAs and the T3 promoter. After linearization, the guide RNAs were transcribed in vitro using RNA polymerase. Similarly, Cas9 RNA transcripts were prepared using the Cas9 vector (Addgene ID: 48625). Fertilized eggs were collected from C57BL/6N mice, and two guide RNAs and Cas9 RNA were injected into the eggs, which were then transplanted into the oviducts of pseudopregnant ICR mice. The genotype of the pups was determined using genomic PCR. Considering the possibility of mosaicism in F0 mice, and to fix mutations, we used one heterozygous F1 mouse as the founder. All mice were 8 weeks to 8 months old.

Mice were purchased from Japan SLC Co. Ltd (Hamamatsu, Japan). All animal experiments were approved by the Institutional Committee for Experimental Animal Care and Use of Shizuoka University and the University of Tokyo, Japan, and carried out in accordance with the Act on Welfare and Management of Animals and the Guidelines for Proper Conduct for Animal Experimentation (Science Council of Japan).

### Fertility evaluation

Ten-week-old male mice were mated with two 8-week-old B6D2F1 WT female mice for 2 weeks. The female mice were checked every day for a vaginal plug and separated if pregnant. Litter sizes were recorded on delivery.

### Histological analysis of testes and sperm

Mouse testes and epididymides were dissected, fixed in Bouin’s solution, and embedded in paraffin. The paraffin blocks were sliced into 4-µm-thick sections, and the sections were then mounted on poly-l-lysine-coated glass slides. The slides were deparaffinized and stained using H&E (hematoxylin and eosin) or periodic acid solution. Images were captured using an IX-70 microscope (OLYMPUS, Japan) equipped with a digital camera (EOS6D; Cannon, Japan). To evaluate the morphology, number, and motility of sperm, the cauda epididymis was incised at a few places and sperm were released into TYH medium by gentle pushing. Subsequently, the sperm were smeared on glass slides. Sperm morphology was monitored under a microscope, the number of sperm was counted using a hemocytometer, and sperm motility was evaluated by visual observation under a stereomicroscope.

### Immunofluorescence analysis of isolated testicular cells and tissue sections

Testes were dissected and then kept in a Petri dish containing phosphate-buffered saline (PBS). Seminiferous tubules were released by removing the tunica albuginea, put into a microtube, and cut into small pieces in PBS using scissors. Cells were mechanically dissociated by pipetting, filtered using a 70 µm nylon mesh, and washed twice with Dulbecco’s modified Eagle medium (DMEM) (D5796; Sigma-Aldrich, St. Loius, MO, USA). The cell suspension was placed on poly-L-lysine-coated glass cover slips in 35 mm dishes and incubated for 30 min in a CO_2_ incubator at 37 °C. Next, the cells were fixed in 3.8% paraformaldehyde (PFA) in PBS, permeabilized using 0.2% Triton X-100/PBS, and blocked using 5% skim milk/PBS for 1 h. Subsequently, the cells were incubated with a primary antibody for 1.5 h, washed with PBS, and incubated with Alexa Flour 488- or Alexa Flour 546-conjugated secondary antibody (Thermo Fisher Scientific, Waltham, MA, USA) for 1 h. Then, nuclei were stained using DAPI. Alexa488-conjugated peanut agglutinin (Thermo Fisher Scientific, Waltham, MA, USA) was used at 10 µg/mL for visualization of acrosome. Tissue sections were deparaffinized and heated for 5 min in citric acid buffer (pH 6.0) using a pressure cooker for antigen retrieval. Immunostaining was conducted similarly. Finally, fluorescence images were captured using an epifluorescence microscope (BX-60; OLYMPUS, Japan) equipped with a digital camera (DP-71; OLYMPUS, Japan or WRAYCAM-NOA630B; WRAYMER, Japan).

### Cell culture and transfection

HEK293F and A549 cells were cultured in DMEM supplemented with 10% fetal bovine serum and penicillin–streptomycin–amphotericin B solution (FUJIFILM Wako Pure Chemical Corporation, Osaka Japan). Vector transfection was performed using Polyehylenimine Max (Polysciences, Inc., Warrington, PA, USA). The 3 × FLAG-tagged human DLEC1 expression vector was constructed by replacing the green fluorescent protein tag in the DLEC1 expression vector (Addgene ID: 90206) to the 3 × FLAG sequence. 3 × FLAG-tagged human DLEC1 was also subcloned into the pCX puro vector. This vector contains internal ribosome entry site sequences that allow the simultaneous expression of DLEC1 and puromycin resistance gene. 3 × FLAG-tagged hDLEC1 containing the pCX vector were transfected A549 cells for establishment of A549 cells stably expressing DLEC1. Cells were selected by 1 µg/mL puromycin for 2 weeks. Immunofluorescence analysis of A549 cells was performed like isolated testicular cells. Images were captured by a digital camera and cilia length was measured using Micro Studio software (WRAYMER, Japan).

### Tubulin polymerization assay

Tubulin polymerization assay was performed, as previously described, with slight modifications^51^. Briefly, HEK293F cells transiently expressing human DLEC1 (transfection efficiency is 60–70%) or mouse testicular cells were cultured in a 35 mm dish, washed with prewarmed (37 °C) PBS, and lysed with hypotonic buffer [20 mM Tric-Cl (pH 6.8), 1 mM MgCl_2_, 2 mM ethylene glycol-bis(β-aminoethyl ether)-*N*,*N*,*N*′,*N*′-tetraacetic acid (EGTA), 0.5% NP-40, 10 mM NaF, 1 mM Na_3_VO_4_, 1 mM phenylmethylsulfonyl fluoride (PMSF)] supplemented with 1 μM paclitaxel at room temperature. The lysate was centrifuged at 17,000×*g* for 10 min at room temperature, and supernatants were put into new tubes and mixed with an equal volume of 2 × sample buffer. The precipitants were also solubilized with 2 × sample buffer, and the samples were incubated for 5 min at 100 °C. Finally, the amount of α-tubulin in each fraction was determined using western blotting.

### SDS-PAGE and western blotting

Tissues or cells were lysed using 1% Triton X-100 lysis buffer [50 mM Tris–Cl (pH 7.4), 150 mM NaCl, 2 mM ethylenediaminetetraacetic acid (EDTA), 2 mM PMSF, 2 mM Na_3_VO_4_, 20 mM NaF, and 1% Triton X-100] or RIPA buffer [50 mM Tris–Cl (pH 7.4), 150 mM NaCl, 0.1% sodium dodecyl sulfate (SDS), 1% NP-40, 0.5% sodium deoxycholate, 2 mM PMSF]. When RIPA buffer was used, the lysate was sonicated to reduce viscosity. Next, the lysate was centrifuged for 20 min at 4 °C, and the supernatant was collected. For immunoprecipitation, cell lysates were incubated overnight with 1 µg anti-FLAG antibody and protein G-agarose beads with gentle agitation at 4 °C. Subsequently, the beads were washed several times. The lysate or beads were then mixed with sample buffer and incubated for 30 min at 37 °C or for 5 min at 100 °C. The proteins were separated with sodium dodecyl sulfate polyacrylamide gel electrophoresis (SDS-PAGE) and transferred onto polyvinylidene fluoride (PVDF) membranes. Finally, western blotting was performed following standard procedures. HRP-conjugated light chain specific goat anti-mouse IgG (Jackson Laboratory, Bar Harbor, ME, USA) was used as secondary antibody to avoid the detection of antibody used for immunoprecipitation, if necessary.

### Electron microscopy

Electron microscopy was performed, as previously described^52^. Briefly, mouse testes were fixed by perfusion with 4% PFA, followed by immersion fixation in 2.5% glutaraldehyde overnight at 4 °C. The testes were then postfixed in 1% osmium tetroxide and embedded in Epon resin 812. Ultrathin sections were cut and stained using uranyl acetate and lead citrate. Finally, the sections were observed under a JEOL JEM-1200 EX transmission electron microscope (JEOL Ltd., Tokyo, Japan).

### Identification of DLEC1-interacting proteins

HEK293F cells transiently expressing 3 × FLAG-tagged hDLEC1 or an empty vector were lysed using 1% Triton X-100 buffer, and insoluble materials were removed by centrifugation. Then, 20 mg of lysates were mixed with 40 µL of anti-FLAG monoclonal antibody-conjugated agarose beads (Sigma-Aldrich) and incubated overnight with gentle rotation at 4 °C. The immunoprecipitants were washed several times and eluted with 2-mercaptoethanol-free 2 × sample buffer for 30 min at 37 °C. The suspension was centrifuged. Next, supernatants were collected, 2-mercaptoethanol was added, and the mixture was boiled for 5 min. Proteins were separated by SDS-PAGE and visualized using Coomassie Brilliant Blue (CBB) staining. The bands of proteins enriched by interaction with hDLEC1 were cut into small pieces. Then, proteins in the gel were reduced using 10 mM dithiothreitol (DTT) and S-alkylated cysteine residues with 55 mM iodoacetamide and digested using 10 ng/mL of sequence-grade trypsin (Promega Corporation, Madison, WI, USA) overnight at 37 °C. Peptides extracted from the gel were analysed using LC–MS/MS with a NanoFrontier eLD linear ion trap time-of-flight mass spectrometer (Hitachi High-Technologies Corporation, Japan) coupled with the NanoFrontier nLC (Hitachi High-Technologies). Briefly, trypsin-digested peptides were separated using a Capillary EX-Nano column (GL Sciences, Japan) and eluted with a linear gradient from 5 to 40% of solvent (98% acetonitrile and 0.1% formic acid) for 60 min at a flow rate of 200 nL/min. Next, the eluent was ionized using a nanoelectrospray ionization source equipped with a SilicaTip (New Objective, Woburn, MA, USA), and MS and MS/MS spectra were obtained in positive ion mode at a scan mass range of *m*/*z* 200–2000. To identify proteins, MS and MS/MS data were analysed using de novo sequencing and protein identification software PEAKS version 7.0^53^. To avoid false positive, we selected proteins with two or more matched peptides and a higher − 10log (*P*-value) (≥ 50).

### Sucrose density gradient centrifugation

We prepared 12 mL of 10–40% sucrose density gradient in a polypropylene tube (331372; Beckman Coulter Inc., Brea, CA, USA) using a gradient maker (SANPLATEC Corp., Osaka, Japan). Testicular cell lysates were layered onto the sucrose gradient with a molecular weight marker (GE Healthcare, Chicago, IL, USA) and centrifuged at 28,000 rpm for 16 h using a SW41Ti rotor (Beckman Coulter) at 4 °C. Then, the fractions were collected from the bottom using a grass capillary connected with a peristaltic pump. Finally, the proteins were precipitated from each fraction using trichloroacetic acid, and the pellets were washed twice with ice-cold acetone, air-dried, and solubilized using 8 M urea.

## Supplementary information

Supplementary Information.
