# Supplementary material for: Dlec1 is required for spermatogenesis and male fertility in mice
Source: Sci Rep. 2020 Nov 3;10:18883. doi: 10.1038/s41598-020-75957-y (PMC7642295; doi:10.1038/s41598-020-75957-y)

Supplementary information

Dlec1 is required for spermatogenesis and male fertility in mice

Yu Okitsu, Mamoru Nagano, Takahiro Yamagata, Chizuru Ito, Kiyotaka Toshimori, Hideo Dohra, Wataru Fujii, and Keiichiro Yogo

Supplemental Tables

Table S1. DLEC1-interating proteins identified by LC-MS/MS analysis.

3×FLAG-tagged hDLEC1 is expressed and immunoprecipitated with anti-FLAG antibody. Co-immunoprecipitated proteins were identified by LC-MS/MS analysis. Band number is as shown in Fig. S5. Accession number, protein name, –10×log10(*P*-value) (–10lg*P*), percentage of protein coverage, peptide number identified, and theoretical molecular weight (MW) are shown.

Band	Accession no.	Protein name	–10lg*P*	Coverage	Peptide	MW
1	NP_005372.2	nucleolin	128.0	10%	8	76,615
1	NP_002799.3	26S proteasome non-ATPase regulatory subunit 2 isoform 1	90.9	6%	4	100,200
2	NP_005336.3	heat shock 70 kDa protein 1A	254.6	37%	25	70,052
2	XP_011541100.1	heat shock cognate 71 kDa protein isoform X1	224.9	34%	21	70,898

2	NP_006100.2	protein arginine N-methyltransferase 5 isoform a	184.7	26%	17	72,684
2	NP_005518.3	heat shock 70 kDa protein 1-like	215.9	21%	15	70,375
2	NP_001008800.1	T-complex protein 1 subunit gamma isoform c	90.7	11%	5	56,431
2	XP_011540543.1	PREDICTED: ATPase family AAA domain-containing protein 3B isoform X2	58.6	5%	2	54,073
3	NP_955472.1	60 kDa heat shock protein, mitochondrial	218.8	40%	19	61,055
3	NP_002697.1	protein phosphatase 1B isoform 1	150.5	32%	9	52,643
3	NP_006576.2	T-complex protein 1 subunit theta isoform 1	148.8	24%	12	59,621
3	XP_011516918.1	heterogeneous nuclear ribonucleoprotein K isoform X4	96.8	13%	5	48,511
3	NP_036205.1	T-complex protein 1 subunit epsilon isoform a	83.9	13%	7	59,671
3	XP_006723200.1	U4/U6 small nuclear ribonucleoprotein Prp31 isoform X1	103.4	11%	5	55,456
3	NP_001753.1	T-complex protein 1 subunit zeta isoform a	86.6	11%	6	58,024
3	NP_002800.2	26S proteasome non-ATPase regulatory subunit 3	69.2	10%	5	60,978
3	NP_110379.2	T-complex protein 1 subunit alpha isoform a	60.8	8%	4	60,344
4	NP_001290045.1	tubulin alpha-1C chain isoform b	205.0	49%	19	46,057
4	NP_001257329.1	tubulin alpha-1A chain isoform 2	200.5	44%	18	46,297
4	NP_006073.2	tubulin alpha-1B chain	199.4	40%	18	50,152
4	NP_006079.1	tubulin beta-4B chain	142.7	28%	10	49,831
4	NP_821133.1	tubulin beta chain isoform b	135.5	25%	9	49,671
4	NP_009202.1	serine/threonine-protein kinase 38	138.5	23%	10	54,190
4	NP_001185771.1	T-complex protein 1 subunit beta isoform 2	114.0	16%	7	52,718
4	NP_006421.2	T-complex protein 1 subunit delta isoform a	109.1	16%	7	57,924
4	NP_001244264.1	ATP synthase subunit alpha, mitochondrial isoform c	97.4	16%	7	54,494

4	XP_011523470.1	dnaJ homolog subfamily C member 7 isoform X1	110.4	13%	5	50,097
4	NP_055815.1	serine/threonine-protein kinase 38-like	100.4	13%	5	54,003
4	NP_006614.2	D-3-phosphoglycerate dehydrogenase	94.5	8%	4	56,651
4	NP_004452.1	phenylalanine—tRNA ligase alpha subunit	85.9	7%	3	57,564
4	NP_002884.1	histone-binding protein RBBP7 isoform 2	64.7	5%	2	47,820
4	XP_011530780.1	T-complex protein 1 subunit eta isoform X1	63.1	4%	2	54,804
5	NP_821133.1	tubulin beta chain isoform b	219.8	43%	23	49,671
5	NP_006079.1	tubulin beta-4B chain	220.0	43%	23	49,831
5	NP_821080.1	tubulin beta-2B chain	216.1	43%	23	49,953
5	NP_006077.2	tubulin beta-3 chain isoform 1	204.0	34%	18	50,433
5	NP_115914.1	tubulin beta-6 chain isoform 1	181.2	33%	16	49,857
5	NP_001308119.1	ruvB-like 2 isoform 2	132.9	21%	8	47,404
5	NP_003132.2	E3 ubiquitin-protein ligase TRIM21	118.0	20%	9	54,170
5	NP_001306013.1	ruvB-like 1 isoform 2	65.5	12%	4	42,127
5	NP_001350501.1	heterogeneous nuclear ribonucleoprotein H isoform b	94.0	11%	4	47,087
6	NP_004550.2	nuclease-sensitive element-binding protein 1	123.9	23%	4	35,924
6	NP_001393.1	elongation factor 1-alpha 1	130.2	22%	11	50,141
6	NP_001530.1	dnaJ homolog subfamily A member 1 isoform 1	114.4	20%	7	44,868
6	NP_001303993.1	methylosome protein 50 isoform 4	97.2	18%	4	29,651
6	NP_001191439.1	eukaryotic initiation factor 4A-I isoform 2	101.6	14%	4	39,548
6	NP_003312.3	elongation factor Tu, mitochondrial isoform 1 precursor	101.7	12%	7	49,875
6	NP_002794.1	26S protease regulatory subunit 7 isoform 1	93.1	10%	4	48,634
6	NP_005871.1	dnaJ homolog subfamily A member 2	89.1	9%	4	45,746

6	NP_001091675.1	heterogeneous nuclear ribonucleoprotein F	60.6	8%	3	45,672
6	NP_000959.2	60S ribosomal protein L4	61.2	6%	2	47,697
7	NP_001243728.1	glyceraldehyde-3-phosphate dehydrogenase isoform 2	100.3	10%	2	31,548
8	NP_000996.2	40S ribosomal protein S3 isoform 1	138.2	31%	7	26,688
8	NP_001164015.1	serine/threonine-protein phosphatase PGAM5, mitochondrial isoform 2	99.6	18%	5	31,876
8	NP_005310.1	histone H1.2	93.7	15%	3	21,365
8	NP_000997.1	40S ribosomal protein S3a isoform 1	73.7	14%	4	29,945
8	NP_002943.2	40S ribosomal protein S2	66.0	14%	4	31,324
8	NP_006089.1	receptor of activated protein C kinase 1	84.5	13%	4	35,077
8	NP_001001.2	40S ribosomal protein S6	88.6	13%	3	28,681
8	NP_001304700.1	60S ribosomal protein L8	65.2	10%	3	28,025
8	NP_006752.1	14-3-3 protein epsilon	52.0	7%	2	29,174
9	NP_001143.2	ADP/ATP translocase 2	135.1	23%	8	32,852
9	NP_001627.2	ADP/ATP translocase 3	111.8	23%	7	32,866
9	NP_001142.2	ADP/ATP translocase 1	102.1	20%	6	33,065
9	NP_000962.2	60S ribosomal protein L7 isoform 1	84.3	18%	4	29,226
9	NP_001269163.1	proteasome subunit alpha type-6 isoform c	80.8	10%	2	25,290
9	NP_000998.1	40S ribosomal protein S4, X isoform	55.1	10%	3	29,598

Table S2. Primer pairs used in the study

Target	Sequence
Dlec1<br>(for Genome PCR)	5′- GAATCAACTTGAGGAGGAAAAGGTTTATTTG -3′ (F2)<br>5′- AAAAGCAGGCAGAAGTTCACAGTTAAG -3′ (R1)
Dlec1<br>(for RT-PCR)	5′- TGTACGCTGAGGTGCAGCAG -3′ (F1)<br>5′- CAGATTCCAGGCCGAATGCG -3′ (F3)<br>5′- CTGGTTCACGAGACAGGTCC -3′ (R2)
Vill<br>(for RT-PCR)	5′- CGACTGTTCGAGTGCTCCAG -3′<br>5′- TTGCTGCTGCTTCCACTTGG -3′
Ctdspl<br>(for RT-PCR)	5′- CAGGTCATTCCCGTACCAAG -3′<br>5′- ACCTGCTACAGAGTCTGTGC -3′
Gapdh<br>(for RT-PCR)	5′- CAGATTCCAGGCCGAATGCG -3′<br>5′- CTGGTTCACGAGACAGGTCC -3′
Off-target 1, Ch10 (for off-target effect analysis)	5′-GCGGATTGGGTAGGTTGTTGTGAGG -3′<br>5′- ACCCTCTGCCCCCTACACTCAACTC -3′
Off-target 1, Ch12 (for off-target effect analysis)	5′- CCTGTGAACCACACAGCCTTCTCC -3′<br>5′- AGAATCGGTGTCAATGGCCCGAGG -3′
Off-target 2, Ch11 (for off-target effect analysis)	5′- CGTGGACCGGACTTTACCAGAGACG -3′<br>5′- AAGGGGAGAGTGCTGCTTTTGCCC -3′
Off-target 2, Ch12 (for off-target effect analysis)	5′- GACCTTGGGCACACTAAGGTGACAC -3′<br>5′- GCCCTTGACAGACGTCCACGGTAG -3′

Table S3. Primary antibodies used in the study

**Antibody (Source, clone)**	**Supplier**	**Cat. no.**	**Concentration (application)**
Dlec1 (Rabbit poly)	Custom-made	-	1 μg ml^{-1} (western blotting)
FLAG (mouse mAb, M2)	Sigma	F1804	1 μg ml^{-1} (western blotting)<br>5 μg ml^{-1} (immunostaining)
KIF3A (Rabbit poly)	GeneTex	GTX11259	2.7μg ml^{-1} (western blotting)
IFT25 (Rabbit poly)	Proteintech	15732-1-AP	0.2 μg ml^{-1} (western blotting)<br>1 μg ml^{-1} (immunostaining)
IFT140 (Rabbit poly)	Proteintech	17460-1-AP	0.1 μg ml^{-1} (western blotting)<br>2.5 μg ml^{-1} (immunostaining)
GAPDH (mouse mAb, 5A12)	WAKO	016-25523	0.05 μg ml^{-1} (western blotting)
α-tubulin (mouse mAb, 10G10)	WAKO	017-25031	1 μg ml^{-1} (western blotting)<br>5 μg ml^{-1} (immunostaining)
α-tubulin (Rabbit poly)	Proteintech	11224-1-AP	0.03 μg ml^{-1} (western blotting)<br>1 μg ml^{-1} (immunostaining)
β-tubulin (mouse mAb, D-10)	Santacruz	sc-5274	0.2 μg ml^{-1} (western blotting)

γ-tubulin (mouse mAb, C-11)	Santacruz	sc-17787	0.1 μg ml^{-1} (western blotting)
TCP-1 α (mouse mAb, B-3)	Santacruz	sc-374088	0.1 μg ml^{-1} (western blotting)<br>0.5 μg ml^{-1} (immunostaining)
TCP-1 β (mouse mAb, (D-8)	Santacruz	sc-374152	0.1 μg ml^{-1} (western blotting)
TCP-1 γ (mouse mAb, F-3)	Santacruz	sc-271336	0.1 μg ml^{-1} (western blotting)
TCP-1 δ (mouse mAb, H-1)	Santacruz	sc-137092	0.1 μg ml^{-1} (western blotting)
TCP-1 ε (mouse mAb, G-3)	Santacruz	sc-376188	0.1 μg ml^{-1} (western blotting)
TCP-1 ζ (mouse mAb, F-4)	Santacruz	sc-514466	0.1 μg ml^{-1} (western blotting)
TCP-1 η (mouse mAb, A-8)	Santacruz	sc-271951	0.1 μg ml^{-1} (western blotting)
BBS1 (Rabbit poly)	Proteintech	21118-1-AP	0.5 μg ml^{-1} (western blotting)
BBS2 (mouse mAb, A-12)	Santacruz	sc-365355	0.1 μg ml^{-1} (western blotting)<br>0.5 μg ml^{-1} (immunostaining)
BBS4 (mouse mAb, 1292CT845.130.218)	Santacruz	sc-517315	0.1 μg ml^{-1} (western blotting)
BBS5 (mouse mAb, B-11)	Santacruz	sc-515331	0.1 μg ml^{-1} (western blotting)
BBS6 (mouse mAb, F-8)	Santacruz	sc-390077	0.1 μg ml^{-1} (western blotting)
BBS7 (mouse mAb, E-8)	Santacruz	sc-390403	0.1 μg ml^{-1} (western blotting)

Supplementary Fig. S1

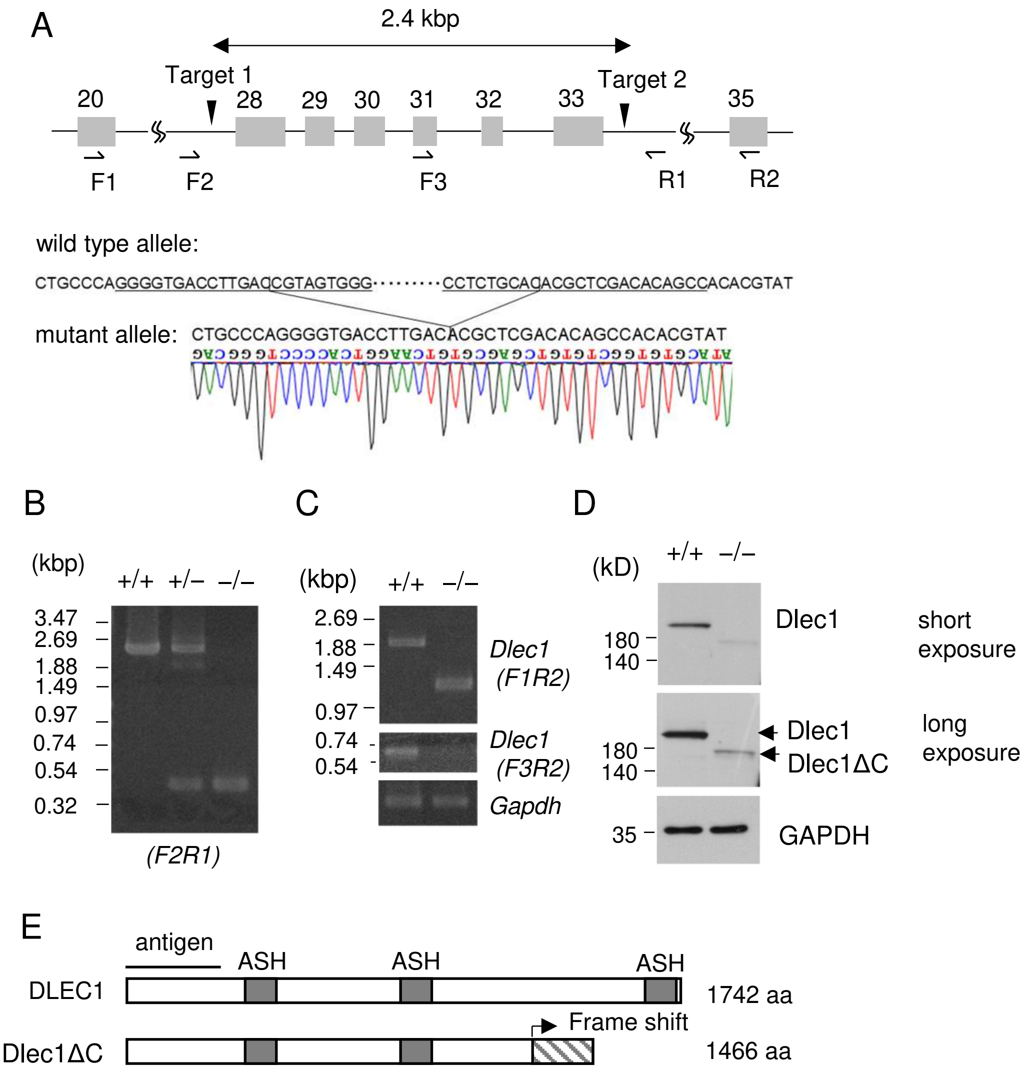

Figure S1. Generation of *Dlec1*-KO mice using the CRISPR-Cas9 system.
(A) Schematic representation of genome editing. Exon of *Dlec1* (gray boxes), two target sites (1 and 2), and primer-binding site used for PCR (F1, F2, F3, R1, and R2) (upper panel). Nucleotide sequence of the WT allele in the vicinity of two target sites (underlined). Deletion of exons 28–33 in the mutant allele was confirmed by sequence analysis (lower panel). (B) Genomic PCR analysis in WT (+/+), heterozygous mutant (+/–), and homozygous mutant (–/–). F2R1 indicates the primer set used. (C) RT-PCR analysis of *Dlec1* in WT and homozygous mutant (–/–) mouse testes. Two primer sets (F1R2 and F3R2) were used. (D) Dlec1 expression in WT (+/+) and homozygous mutant (–/–) mouse testes was analysed by western blotting. (E) Structure of mouse DLEC1 protein expressed in WT and homozygous mutant mice. ASH, ASPM-SPD-2-Hydin domain. Dlec1ΔC lacks one ASH domain and has an extra sequence created by a frameshift mutation in the C-terminus (hatched box). The region for binding the custom-made antibody is also shown. Images of full-length gels and immunoblots are presented in the supplementary Fig. S10.

Supplementary Fig. S2

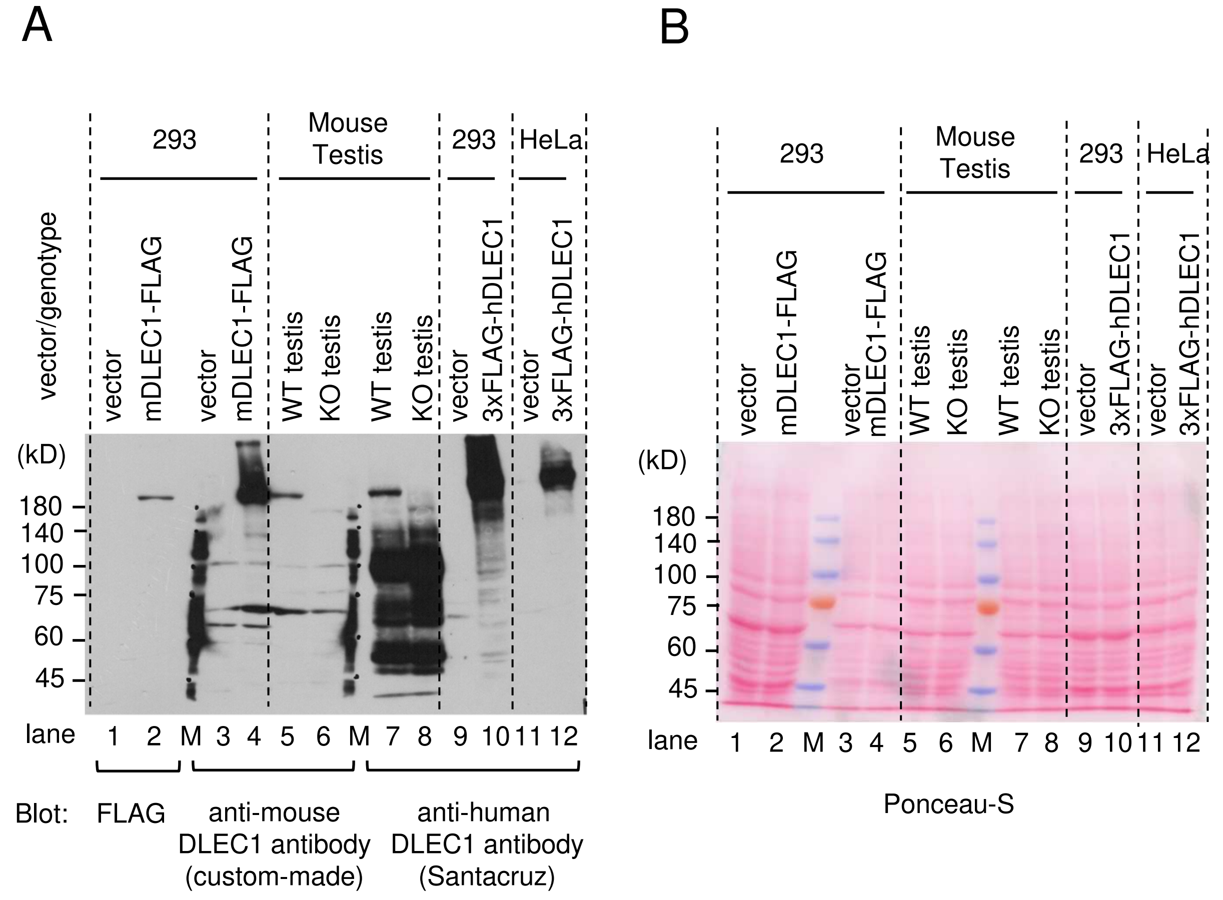

Figure S2. Validation of custom-made anti-mouse DLEC1 antibody.

A) Indicated mouse or human DLEC1 expression vectors were introduced into 293 cells and HeLa cells, and expression of DLEC1 were monitored by western blotting using anti-FLAG antibody (lane 1, 2), custom-made anti-mouse DLEC1 antibody (lane 3, 4), and commercially available anti-human DLEC1 antibody (lane 9–12). Expression of DLEC1 in WT and KO mouse testes were also monitored using anti-mouse DLEC1 antibody (lane 5, 6), and anti-human DLEC1 antibody (lane 7, 8). The antigen of anti-human DLEC1 antibody is 320–349 aa of human DLEC1. The amino acid sequence has 80% homology to the mouse protein. Thirty μg (lane 1–2, 5–12) or 10 μg (lane 3, 4) of protein were loaded to each lane. Our custom-made antibody recognized both exogenously and endogenously expressed DLEC1. B) Membrane stained with Ponceau-S to confirm the amount of protein loaded. M indicates lanes of the protein marker.

Supplementary Fig. S3

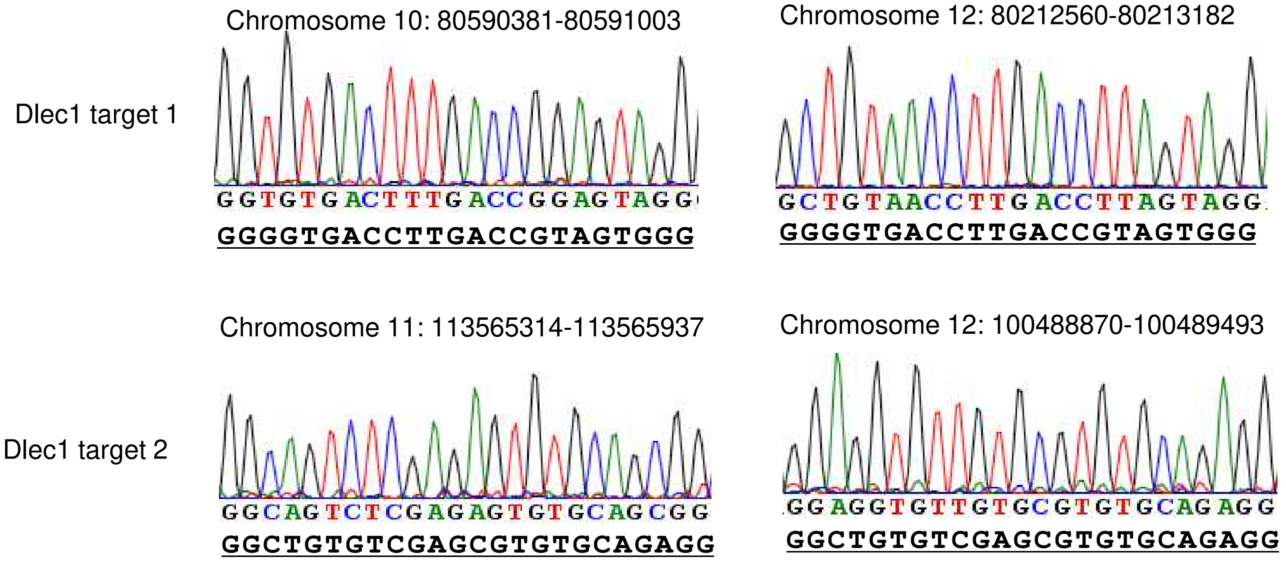

Figure S3. Mutations did not occur in potential off-target sites.
Potential off-target sites for each target sequence (underlined) were searched using TagScan (http://ccg.vital-it.ch/tagger/tagscan.html). Two sites with high similarity were selected and the region amplified by PCR. The nucleotide sequence of PCR products was analysed by direct sequencing. The chromosome number and region of the sites are indicated above waveform data. There were no deletions or insertions in all sites.

Supplementary Fig. S4

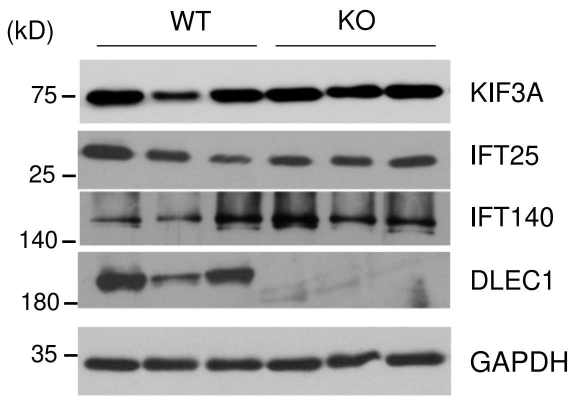

Figure S4. KIF3A and IFT complex subunit expression in *Dlec1*$^{-/-}$ testis.
Testes were collected from WT and *Dlec1*$^{-/-}$ mice (*n* = 3 each), and KIF3A, IFT25, and IFT140 expression was monitored by western blotting. Images of full-length immunoblots are presented in the supplementary Fig. S11.

Supplementary Fig. S5

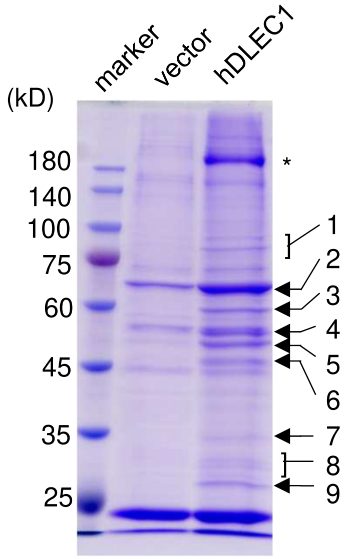

Figure S5. Searching for DLEC1-interacting proteins.
The empty vector (vector) or 3×FLAG-tagged hDLEC1 was expressed in HEK293F, and DLEC1 was immunoprecipitated with anti-FLAG antibody–conjugated sepharose beads. The immunoprecipitant was separated using SDS-PAGE and visualized by CBB staining. *hDLEC1 band. The bands indicated by arrows were cut out and subjected to LC-MS/MS. Identified proteins in each band are listed in Table S2.

Supplementary Fig. S6 (full-length images to generate Fig.1)

Fig. 1A

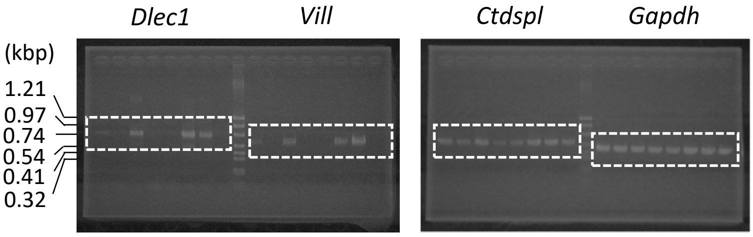

Fig. 1B

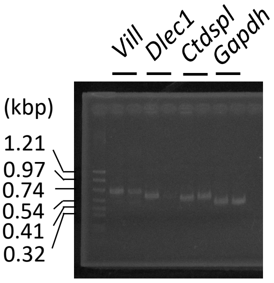

Fig. 1C

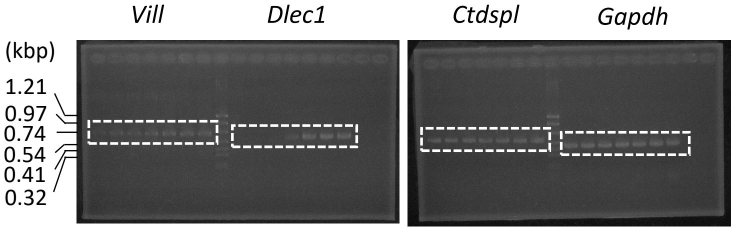

Fig. 1D

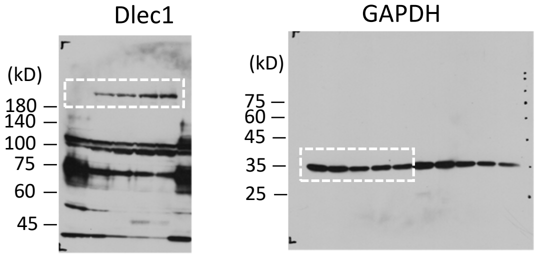

Fig. 1E

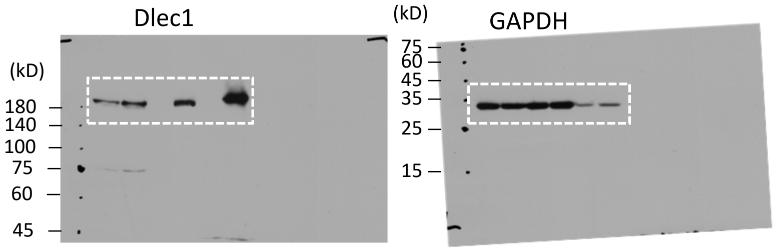

Supplementary Fig. S7 (full-length images to generate Fig.5)

Fig. 5A

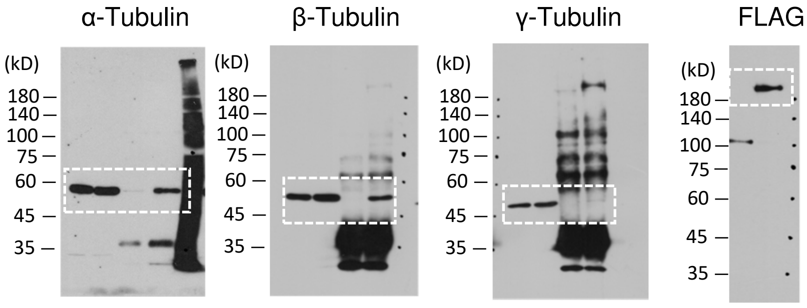

Fig. 5B

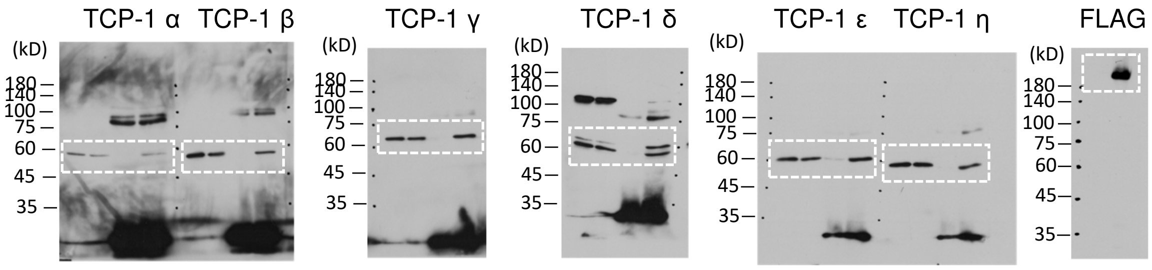

Fig. 5C

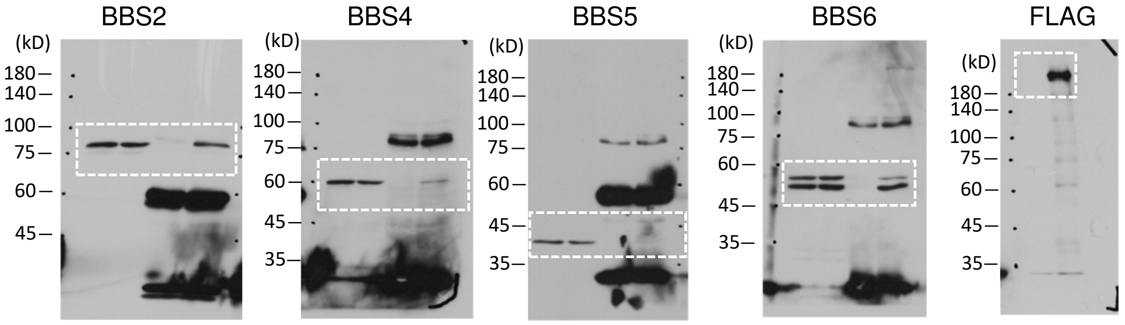

Supplementary Fig. S8 (full-length images to generate Fig.6)

Fig.6A

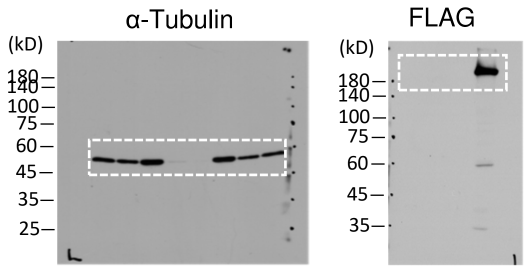

Fig.6C

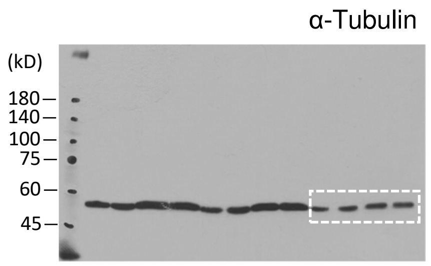

Fig.6D

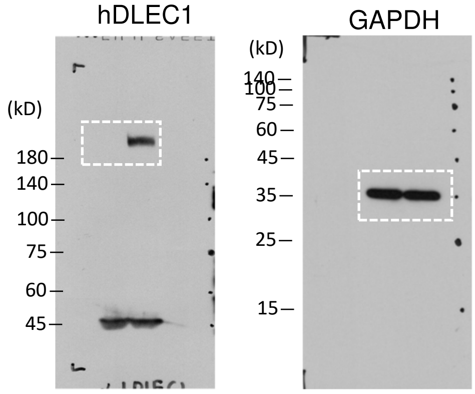

Supplementary Fig. S9 (full-length images to generate Fig.7)

Fig.7A

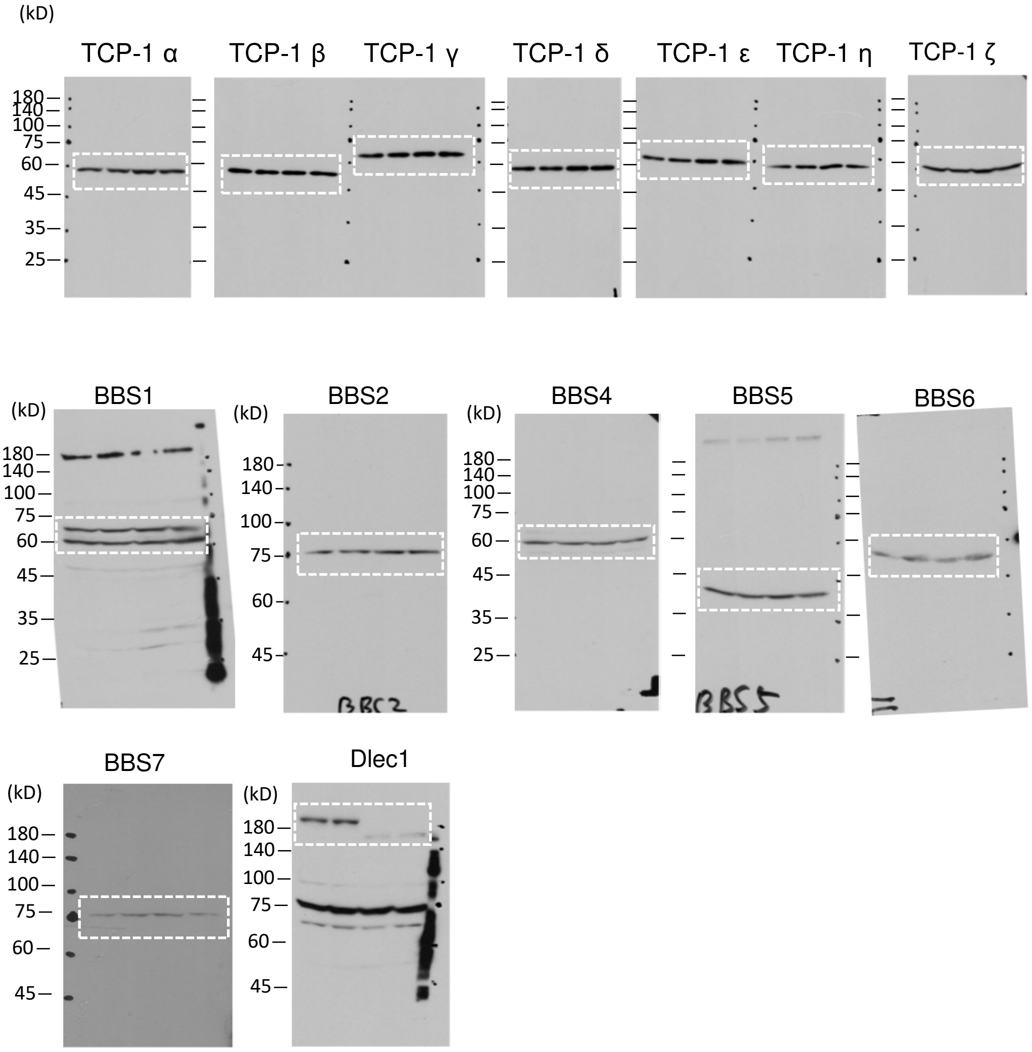

Supplementary Fig. S9 (continued)

Fig.7B

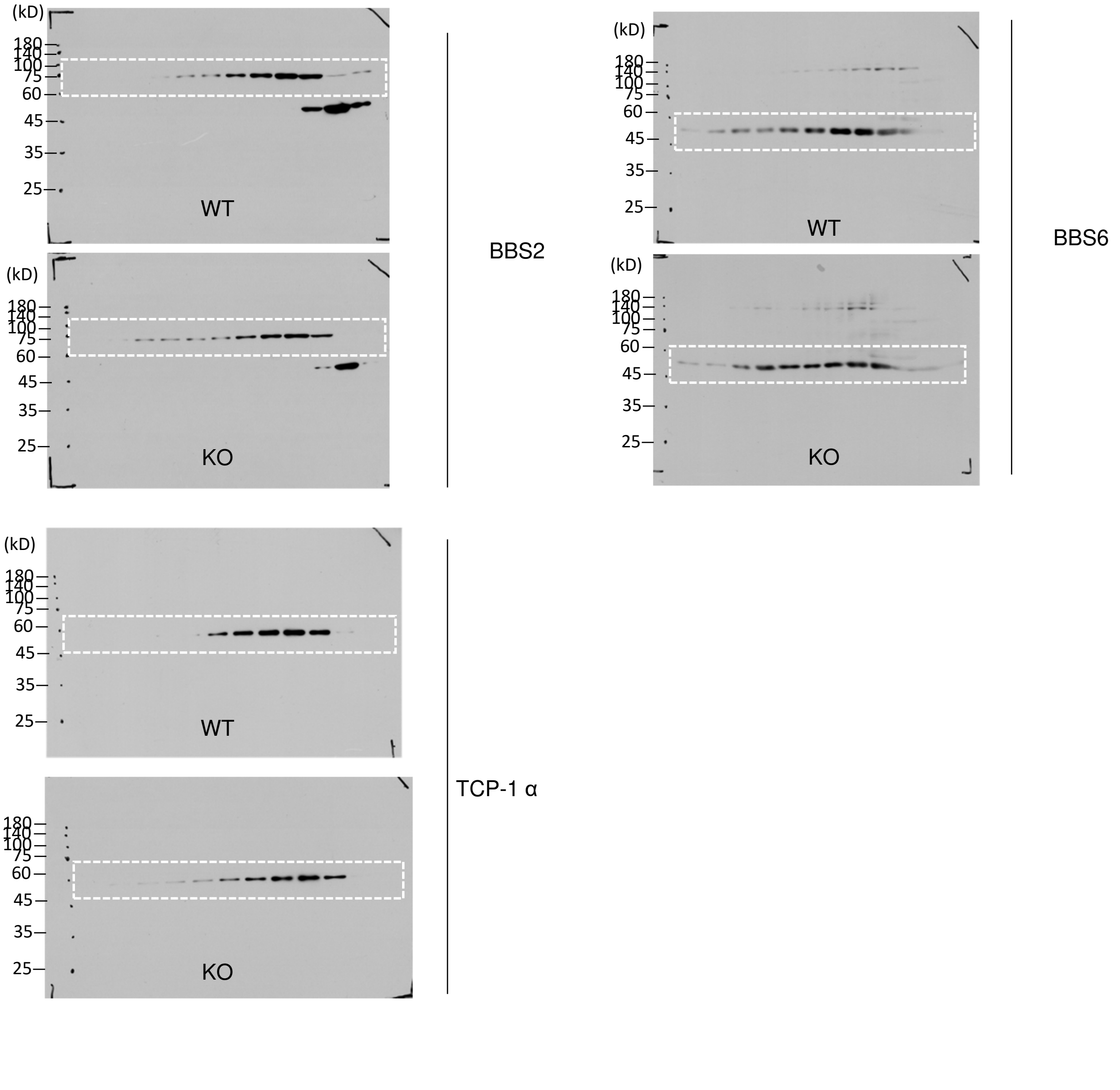

Supplementary Fig. S10 (full-length images to generate supplementary Fig.S1)

Fig. S1B

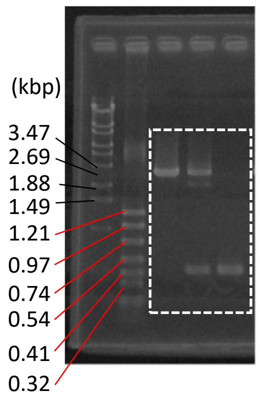

Fig. S1C

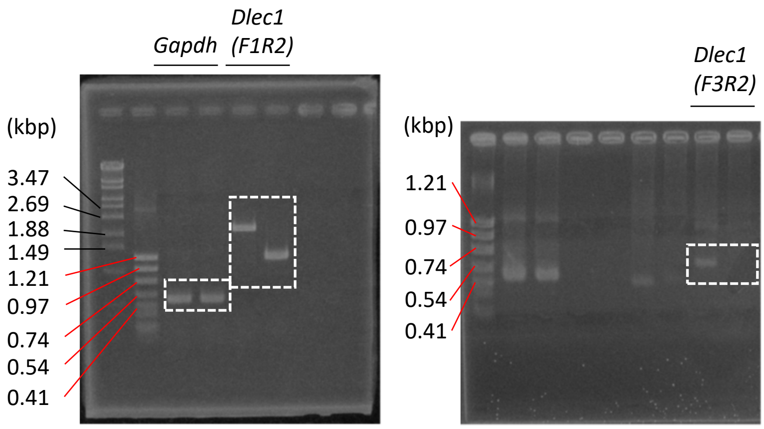

Fig. S1D

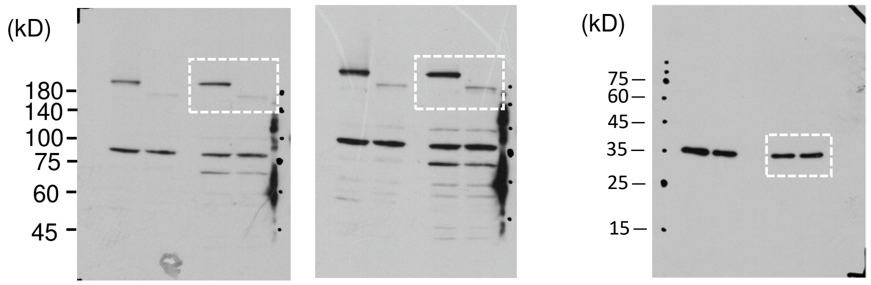

DLEC1 (short exposure)

DLEC1 (long exposure)

GAPDH

Supplementary Fig. S11 (full-length images to generate supplementary Fig.S4)

Fig. S4

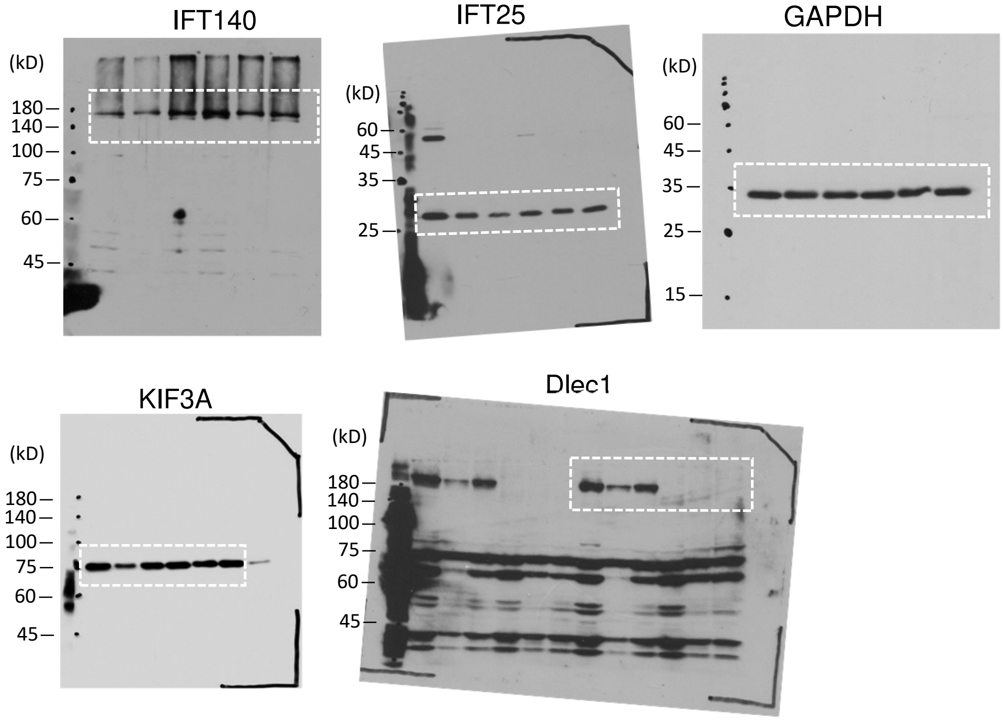

Supplement: Supplementary file 1 — Supplementary Information. [file 41598_2020_75957_MOESM1_ESM.pdf]
